# Experimental Analysis of the Sound-Absorbing Properties of Reused LDPE Bubble Wrap Packaging for the Sustainable Acoustic Requalification

**DOI:** 10.3390/polym18202460

**Published:** 2026-10-09

**Authors:** Giuseppe Ciaburro, Virginia Puyana-Romero

**Affiliations:** 1 Faculty of Engineering and Informatics, Department of Engineering, Pegaso University, 80143 Naples, Italy; 2 Departamento de Ingeniería en Sonido y Acústica, Universidad de Las Américas, Quito 17513, Ecuador; virginia.puyana.romero@udla.edu.ec

**Keywords:** bubble wrap, sound absorption, reused packaging materials, acoustic correction, sound adsorption coefficient

## Abstract

This study examines the acoustical properties of reused bubble wrap, an LDPE bubble wrap packaging material, as a new sound absorber material. In total, seven different structural designs were experimentally examined through manipulating the number of membrane layers, the thickness of the air cavity, the addition of honeycomb cores, and a unique design of a skein (rolled) configuration to obtain a multilayered structure with interconnectivity of air cavities. The normal incidence Sound Absorption Coefficient (SAC) was measured using an impedance tube as per ISO 10534-2:2023. The experimental data show that the acoustical behavior depends on the structural design and its resonance mechanisms. The best SAC achieved was 0.91 for double-layer and 0.97 for triple-layer membrane structures. Honeycomb cores do not improve the maximum sound absorption coefficient, but they expand the frequency range with high absorption levels above 0.63 in the range of 630 to 1500 Hz. The rolled configuration showed the best broadband performance by having absorptions larger than 0.50 over the 315–1500 Hz frequency range, with a peak value of 0.83 at 440 Hz. A Physics-Informed Conditional Neural Network (PICNN) is used to predict the sound absorption coefficient by incorporating physical constraints on the propagation of acoustic waves. The proposed approach successfully predicted the experimental trend by getting R^2^ = 0.9905, MAE = 0.0166, RMSE = 0.0242, and NRMSE = 2.49%. The proposed method not convert bubble wrap waste into a useful and eco-friendly acoustic material but also has great potential for designing and optimizing recycled acoustic metamaterials.

## 1. Introduction

The problem of sustainable management of packaging waste is one of the most urgent issues in both environmental protection and industry in the 21st century. Growing consumption rates, development of e-commerce, and the widespread introduction of delivery services using single-use packaging have resulted in a rise in plastic waste production to a previously unseen level [1]. In spite of notable achievements in the development of recycling technologies and separation of waste collection, a considerable part of packaging produced still gets recovered insufficiently.

The European Union has seen a growing trend of the creation of packaging waste over the last decade, demonstrating the extent to which today’s consumption remains dependent on resources that have a high impact on the environment [2]. In such a situation, there is a challenge to implementing the goals of the European Green Deal and Circular Economy Action Plan since waste prevention, materials reutilization, and resource efficiency become crucial for the decarbonization of the production process [3]. With regard to the discussed challenges, the recently introduced European Packaging and Packaging Waste Regulation (PPWR) sets a stricter legislative framework for the process, with the aim of gradually decreasing the volume of packaging waste produced [3]. To be more specific, the regulation sets mandatory reduction goals of 5% by 2030, 10% by 2035, and 15% by 2040 compared to the base year, at the same time encouraging recycling, reuse, and the introduction of production models in accordance with the circular economy approach.

One of the materials heavily impacted by the described shift is bubble wrap, which is mainly composed of low-density polyethylene (LDPE) [4]. Due to the excellent characteristics of low weight, flexibility, water resistance, and cushioning, bubble wrap has become one of the most commonly used protective packaging materials in the logistics industry and e-commerce shipments. The explosive development of e-commerce leads to even more consumption of this material, making bubble wrap a part of the post-consumer plastic waste generation.

Although LDPE has theoretical recyclability, recycling of bubble wrap is associated with multiple operational difficulties. Being light, taking too much space per kilogram, getting contaminated with other materials, and having expensive collection and transportation makes bubble wrap recycling inefficient. Therefore, a significant amount of this material is going to landfill, is incinerated, or littered in the environment, causing the problem of land- and marine-based plastic waste pollution [5]. As shown by the latest international estimations, most of the plastic packaging is not recycled, making it necessary to create new approaches to the recovery of such materials before disposal.

Here, reuse is one of the best ways provided by the European waste hierarchy, since it allows maintaining the functionality of the material without going through the transformation that occurs during mechanical or chemical recycling processes, which are known for their energy and resource consumption [6]. Particularly, upcycling includes using materials which have reached their initial end of life to create valuable technical objects, thus increasing their life span while saving resources, emitting fewer greenhouse gases, and producing less waste.

One of the promising fields of the application of upcycling can include acoustical engineering of buildings and sustainable building renovation. The rise in the need for environmentally friendly materials for noise reduction has led to extensive studies of recycling of by-products and the search for possible uses of recycled materials, such as natural fiber, recycled textiles, agricultural wastes, lignocellulosic residues, and post-consumer polymer materials [7]. However, despite the wide use of bubble wrap with unique geometric features represented by a regular arrangement of sealed air cells, its acoustic properties remain unstudied.

A study of the acoustical absorption characteristics of bubble wrap would thus allow us to gain different viewpoints in terms of the possible creation of novel, efficient, and environmentally friendly solutions in the field of acoustic renovation of living spaces. The possibility of using a widespread material in the form of a functional component for noise reduction will not only decrease its impact on the environment but also contribute to the creation of design models in accordance with the principles of the circular economy by turning plastic waste into a technical material [8].

The sound environment of the dwelling places has a direct influence on the wellness and good state of residents, which has long been underestimated in designing residential buildings. Sound discomfort must be viewed as equally important as thermal discomfort: the effect of a 3.9 dB increase in the level of noise on people’s comfort is about equal to the effect of a 1 °C increase in the temperature of the operating environment at temperatures varying between 23 and 29 °C [9]. Unreasonably high reverberation times, excessive background noise, and insufficient speech intelligibility affect concentration and stress people, decreasing their productivity. Excessive reverberation and high background noise stimulate the nervous system of the person, raising stress and fatigue. Distraction and poor cognitive activity in the case of open offices or educational spaces are caused by a lack of proper sound management. Modern residential buildings with reflective materials, high ceilings, and open spaces are particularly vulnerable to these problems, which makes acoustic correction an increasingly necessary condition, even at home [10].

Indeed, scientific literature confirms that recycled or waste materials prove effective in use as sound absorbers [11]. The application of recycled materials as an alternative to natural and artificial materials is a real prospect for all industries, which helps reduce waste production and the use of non-renewable resources. Research on sound-absorbing properties of materials obtained from recycled plastic bottles and reclaimed asphalt has shown significant results. For example, Murugan et al. [12] proved that two-stage compression molding of waste polyolefin packaging materials with plasticized aluminum foil, expanded polystyrene, and plant fibers has sound absorption capacity similar to glass wool. With regard to recycled polymeric materials, modern scientific research proposes the use of open-cell foams based on waste microplastics, such as PET and polystyrene, embedded in biopolymer matrices.

Recent research in the field of acoustic membranes has revealed the possible use of thin, flexible foils along with air-filled cavities to improve acoustic efficiency over wide ranges of frequencies due to the synergy of foil resonance and cavity dynamics [13].

Apart from the recycled porous materials and composites, a few studies have been conducted on polymer-based materials in different acoustic designs. For instance, polyolefin foam and closed-cell polyethylene-based foams have been studied for applications in sound absorption and insulation, and it was shown that cellular structure and design could be of acoustic relevance [14]. In addition, polyethylene films have been used in multi-layered sound absorbers, where the effect of films on the performance of the sound absorbers is dependent on the properties and thickness of the surrounding layers [15]. More recently, recycled polymer waste has been mixed with specially designed acoustic metamaterial structures for enhancing low- and mid-frequency absorption [16]. These experiments clearly demonstrate acoustic properties of polyethylene-based materials, polyethylene films, materials obtained from packaging, and engineered polymeric structures. Nonetheless, they mostly address foams, films used in multi-layered absorbers, processed waste-based composites, or specially designed metamaterials, but not commercial bubble wrap as such.

The reported absorptive characteristics of such materials depend greatly on the material structure, thickness, density, backing arrangement, and testing procedure. Porous and cellular polymer structures usually show broadband absorption that is related to the viscous and thermal dissipation effects, while the membrane and cavity absorbers may demonstrate some absorption peaks at certain frequencies. For this reason, the absorption coefficient values cited in the literature cannot be compared directly without knowing the configuration of the sample tested and the test conditions. This comparison is given in the Section 3 below.

These further advancements have led to more design opportunities through the application of the layered structured design approach. The combination of layered composite structures of recycled plastic micro-fiber pulp with acoustic metamaterials has been found to be a highly effective and eco-friendly means of absorbing low-frequency sounds. Significant potential was also discovered in the use of recycled cellulosic materials: the recycled paper fibers, measured using the impedance tube according to ISO 10534-2, produced an average noise reduction coefficient (NRC) value of 0.50, achieving maximum values of 0.98 between 1575 and 1675 Hz [17].

Whereas the body of research relating to sound absorption materials including recycling, natural materials, polymer foams, membranes, composites formed from packaging waste, and acoustic metamaterials has been fairly well studied, the acoustic properties of commercially available low-density polyethylene (LDPE) bubble wrap have not been investigated systematically in comparison to other sound absorbing materials. Specifically, the effects of layer thickness, air cavity depth, and construction have not been well described for bubble wrap as an acoustic material.

It may seem especially important in the context of the widespread usage of this material in the packaging industry and problems related to its recycling through traditional recycling processes. Bubble wrap is made in the form of a polymer film that includes a number of sealed air cells. Considering the specific construction of the bubble wrap cells, some physical phenomena that occur due to the interaction of sound waves and the bubble wrap can be the basis for acoustic behavior, including those related to cavities, thermoviscous losses due to air and polymer interfaces, and viscoelastic losses in polymeric layers. However, all these phenomena need special experiments in order to be investigated separately.

Therefore, the research gap in this work is not only associated with the lack of acoustic data on bubble wrap. The main scientific challenge is to understand how the unique structure of this material, made up of LDPE films and air cavities, impacts sound absorption depending on the material configuration. From the engineering perspective, it is vital to understand whether layering, air-cavity depth, and installation can make this lightweight packaging material effective as a sound absorber. Hence, in addition to proving that bubble wrap can absorb sound, this research aims to discover which structural characteristics and installation parameters impact the acoustic performance of the material.

As mentioned above, based on the discussed acoustic mechanisms, four experimental hypotheses are proposed. The first hypothesis is that adding bubble wrap layers will affect the effective acoustic impedance of the system, which could lead to an increase in peak absorption and the absorption band. The second hypothesis is that increasing the air cavity depth will shift the resonant frequency to lower values according to the properties of a cavity-backed absorber. The third hypothesis is that adding the honeycomb material will have an impact on the resonant effect due to adding more confined air and interaction areas. The last hypothesis is that the rolled configuration will have a more distributed response, which could broaden the absorption band compared to the planar configuration.

This way, the current study explores the sound absorption properties of recycled bubble wrap as a function of frequency and installation configuration. Specifically, the influence of the number of layers, air-cavity depth, and mounting system on the acoustic performance is studied. At the same time, Physics-Informed Conditional Neural Networks (PICNNs) are designed to reveal the link between the mentioned characteristics and sound absorption. The PICNN model contrasts with the conventional neural network approach, which learns the statistical dependencies directly from the experimental data; the presented PICNN uses the physical constraint in its learning process. The intention behind the developed approach is to increase the physical plausibility of the predicted acoustic response, especially when the experimental dataset is relatively small. Nonetheless, a direct quantitative comparison between the two approaches was not within the scope of the current work. In general, the paper offers a quantitative analysis of the acoustic performance of bubble wrap and discusses its possible application in acoustic retrofitting of houses.

## 2. Materials and Methods

### 2.1. Material Description: Bubble Wrap

Bubble wrap is among the most widely used flexible packaging materials, valued for effectively protecting fragile goods during handling, transportation, and storage. Its lightness, deformability, and shock-absorbing capacity make it inexpensive and highly versatile across industrial and commercial applications. Growing e-commerce and delivery demand has increased both material consumption and end-of-life plastic waste. Structurally, bubble wrap consists of two or more polymer films heat-sealed together, enclosing a regular array of air bubbles that function as micro compression chambers dissipating impact energy [18]. Bubble diameters typically range from 10 to 25 mm with heights of 3–8 mm; the uniform cavity distribution gives the material near-isotropic in-plane mechanical properties and high flexibility. The primary material is low-density polyethylene (LDPE), a thermoplastic polyolefin (recycling code #4) produced through ethylene polymerization, forming long (–CH_2_–CH_2_–)_n_ macromolecular chains [19]. LDPE is manufactured via free-radical polymerization under high pressure (1000–3000 bar) and temperature (80–300 °C), producing extensive short and long chain branching that prevents efficient molecular packing and results in relatively low crystallinity (50–60%) [20]. This particular structure differentiates LDPE from high-crystalline polyethylenes such as HDPE, where low crystallinity leads to reduced elastic modulus and stiffness but increased ductility, elastic deformability, and resistance to crack propagation. Low-density polyethylene has good moisture resistance, chemical inertness, impermeability, and easy processing, hence its extensive use in packaging foods and other products in protective films, flexible containers, and bubble wrap [21].

The manufacturing process for bubble wrap requires multiple steps. LDPE pellets undergo melting and extrusion through the process of blown film extrusion. This produces very thin and uniform films that are vacuum formed against the surface of a metal cylinder with cavities that correspond to the bubbles’ design, causing plastic deformation into microchambers. Next, one film is joined to another film through heating, thus encapsulating air inside bubbles and producing a honeycomb structure. LDPE can be mixed with linear low-density polyethylene (LLDPE) to enhance puncture resistance and improve stability of bubbles during the forming process. The functional aspect of bubble wrap may be considered as an acoustically relevant composite made of thin layers of polyethylene film and air bubbles. Though originally engineered for energy dissipation upon impact, it may have acoustic relevant properties due to repeated reflection of sound waves, damping of the trapped air due to viscosity, and deformations of the elastic polyethylene film [22]. The combination of the compressibility of the air and LDPE elasticity may enable similar vibro-acoustic phenomena in bubble wrap as in vibrating panels and cellular materials. Nevertheless, its acoustic properties have not been studied yet, and there is no systematic characterization of bubble wrap as an acoustic absorber available. The environmental challenge associated with bubble wrap is quite obvious. Although it can be recycled using LDPE film supply channels, due to the low density and high mass-per-volume ratio, collection, transportation, and compacting of this waste is rather expensive, resulting in landfilling and incineration of the waste without recovery of the raw material.

Table 1 summarizes key physical, chemical, and mechanical properties of LDPE used in bubble wrap production, drawn from relevant technical and scientific literature [23].

**Figure 1:**
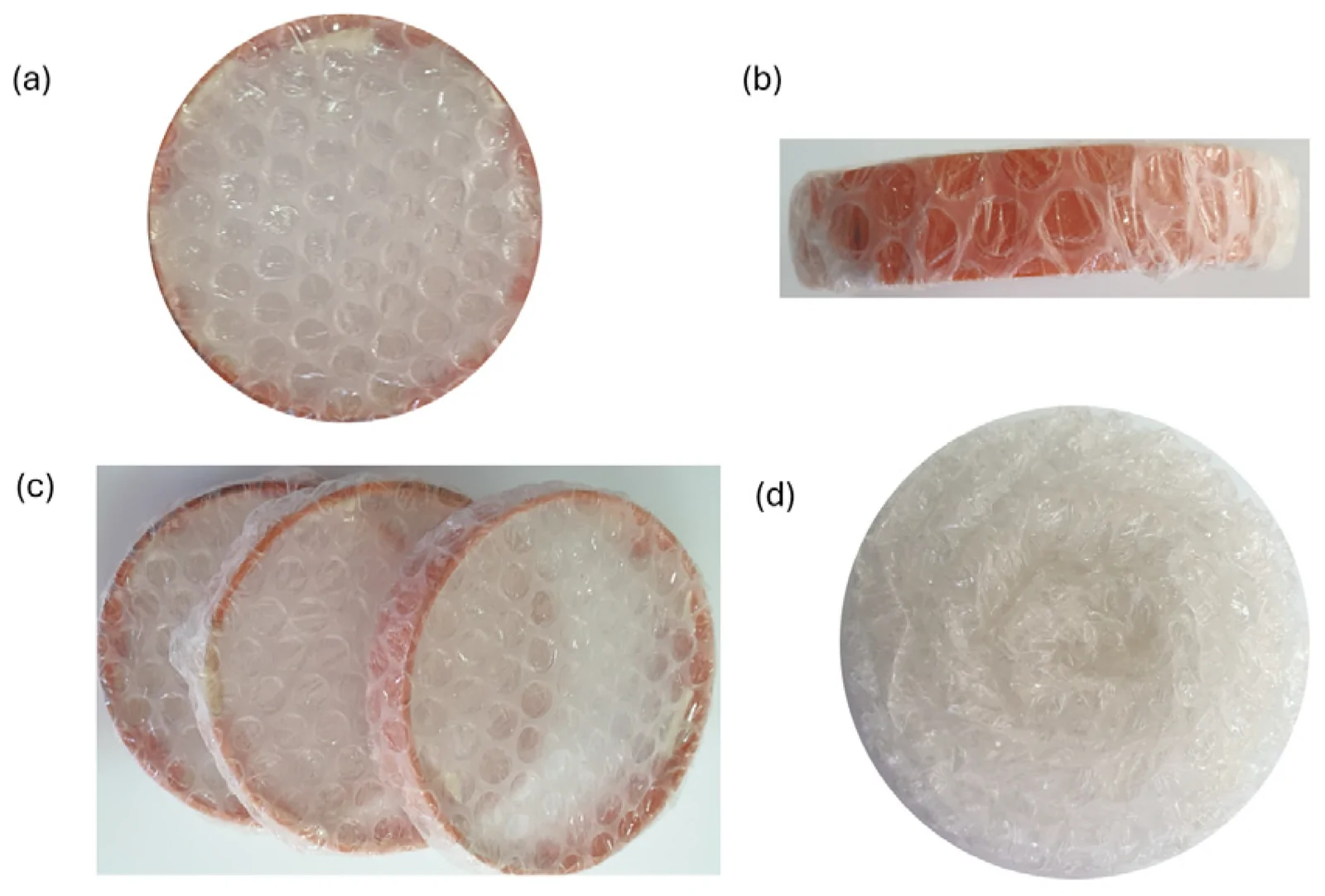
Preparation and assembling of the tested bubble wrap specimens: (**a**) a single bubble wrap specimen attached to a solid PVC base; (**b**) the bubble wrap specimen attached to the PVC base for sound absorbing measurements; (**c**) a multi-layer assembly made of three bubble wrap membranes; (**d**) the rolled bubble wrap specimen making up a coil structure with several folded layers and enclosed cavities.

The samples of bubble wrap used in this study were obtained from actual packaging waste materials and were neither exposed to any chemical treatment nor underwent any cleaning or modification process in order to explore the potential of reuse of this material within the circular economy framework (Figure 1). Thus, the presence of any possible surface contamination linked to prior use cannot be completely ruled out. Nevertheless, no signs of any contamination or damage were detected during visual examination. Commercial packaging material having a nominal bubble size of 10 mm was chosen for the experiment. It consisted of two LDPE films that were bonded by heat bonding and had a thickness of around 100 μm, while the overall thickness of 4–5 mm was due to the presence of air bubbles inside it.

The selected material was in excellent physical condition with no perforations or damaged or collapsed cells. Prior to preparing the specimens, visual inspection of the bubble structure was conducted in order to examine its regularity and uniformity. No damage to the air cells was observed in the specimens used for acoustic measurements. The physical and thermophysical properties of LDPE are listed in Table 1. The geometry of air bubbles was presumed to greatly affect the acoustic characteristics of the material and was estimated through determination of the sound absorption coefficient. Samples were tested in several configurations to examine their influence on sound absorption. Bubble wrap was fastened to a rigid PVC plate to keep it tight (Figure 1a,b).

Multilayer samples were then produced using single or multiple bubble wrap layers combined with air cavities of specified thickness, and in some configurations, honeycomb-shaped internal structures (Figure 1c). These assemblies were developed to investigate the influence of the increasing complexity of the structure on the process of absorption in the low-frequency region. Moreover, a special assembly of the sample was prepared by folding the bubble wrap membrane into a coil structure, resulting in a compact assembly with several folded layers and enclosed cavities (Figure 1d). All samples were prepared with a definite thickness to provide a comparable investigation of the assemblies.

The honeycomb material employed for the experimental setup was an existing commercial product that consisted of aramid paper. The honeycomb had a nominal thickness of 13 mm and hexagonal cells of side length 4 mm. This honeycomb was used in its commercial form without any other modifications of its structure. The geometrical parameters of the honeycomb were kept constant during the experiment, while the diameter and number of wraps were held constant for the rolled form. Any deliberate additional compression of the honeycomb or bubble wrap was not done.

### 2.2. Measurement of the Sound Absorption Coefficient

**Figure 2:**
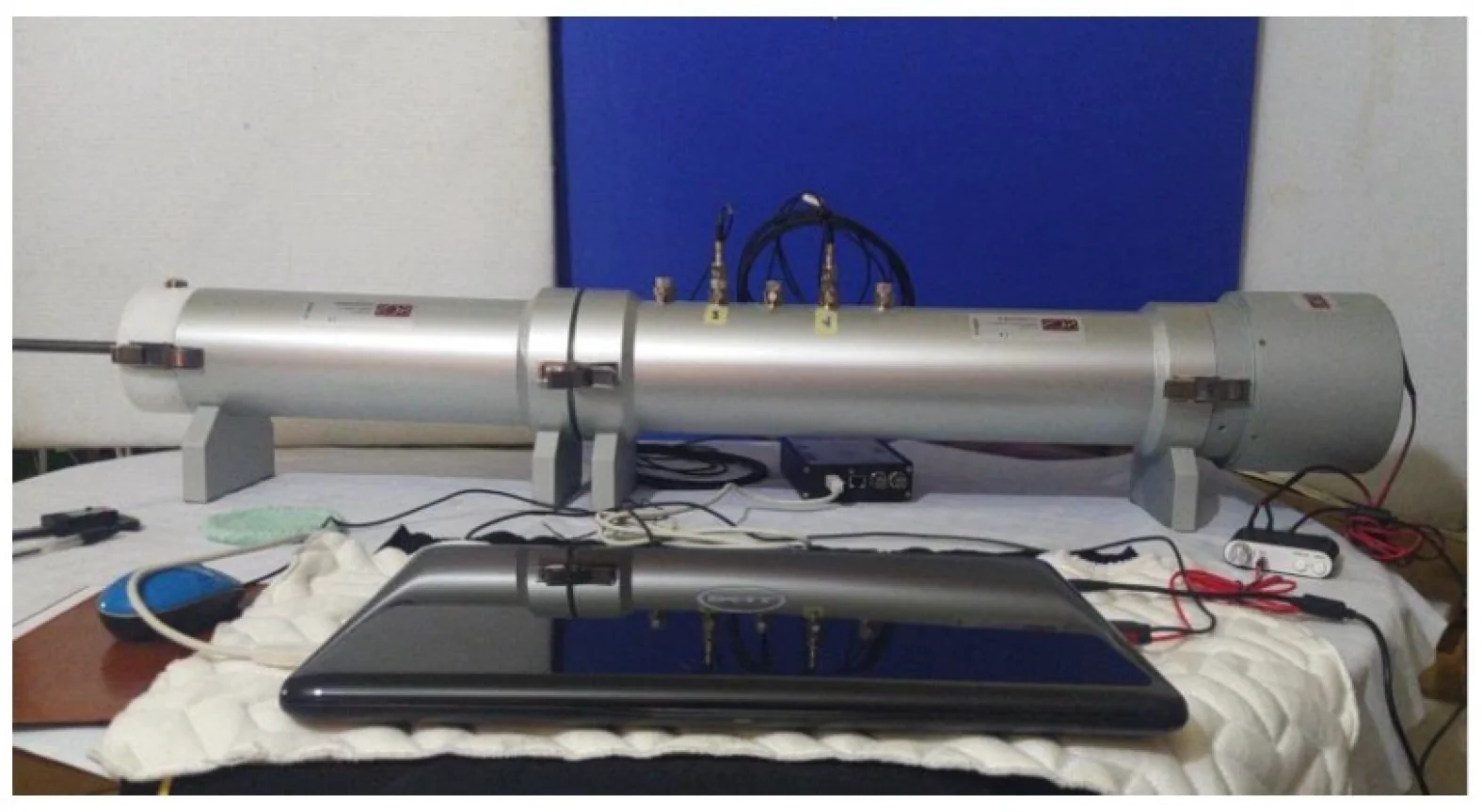
Experimental setup illustrating the impedance tube used (Type 9020 B/K, Model SCS) in determining the sound absorption coefficient of the samples under investigation in normal incidence.

The Sound Absorption Coefficient (α) is a dimensionless parameter (0–1) representing the ratio of absorbed to incident sound energy, where α = 0 indicates perfect reflection and α = 1 indicates complete absorption—making its frequency-dependent measurement the fundamental acoustic test for sound-absorbing materials. Tests followed ISO 10534-2:2023 [24], which specifies an impedance-tube method using two microphone positions and frequency analysis to determine normal-incidence sound absorption coefficients (Figure 2).

This 2023 revision updates the 1998 standard, extending characterization capabilities to include characteristic impedance, dynamic wavenumber, dynamic mass density, and dynamic compressibility modulus for homogeneous materials. The method generates plane waves within the tube via a noise source; sound pressures measured at two fixed wall-microphone positions enable decomposition of the interference field and calculation of the complex acoustic transfer function, normal-incidence absorption coefficient, and material impedance ratios [25]. This transfer function approach is substantially faster than the stationary-wave method in ISO 10534-2 [24]. Unlike the reverberation chamber method, which determines diffuse-incidence absorption under ideal conditions, the impedance tube requires only small samples matching the tube cross-section, making it well-suited for research applications with limited material availability. Notably, normal-incidence and diffuse-incidence coefficire not directly comparable due to differing excitation conditions. Measurements here used the two-microphone Transfer Function Method (TFM).

The experiment setup comprises the following major components:

• A rigid circular steel tube with an internal diameter of 100 mm, ensuring the propagation of only plane waves in the frequency range of interest;
• A loudspeaker mounted at one end of the tube acts as a noise source, fed with a broadband signal (white or pink noise);
• Two condenser microphones mounted on the wall at fixed positions, with mutual distances optimized for the frequency range being analyzed;
• A multichannel signal analyzer for simultaneous acquisition of the signals from the two microphones and calculation of the complex transfer function H_12_;
• A sample of the material is placed inside a dedicated sample holder that is located in the tube end, sealed by a hard piston.

The upper frequency limit of analysis for the impedance tube is related to the diameter of the tube and the microphone spacing [26]. In the case of 100 mm diameter tubes, the upper frequency limit is defined by the fact that the wavelength should exceed the tube diameter. Nevertheless, in tests based on ISO 10534-2, the upper operating limit is estimated more conservatively. In order to ensure the propagation of plane waves only and reduce the impact of higher modes, the requirement is usually:

(1) λ ≥ 2d,

which leads to:

(2) $$f_{\max}=\frac{c}{2d},$$

obtaining:

(3) *f_max_* ≈ 1715 Hz,

The measurements were performed in the frequency range of 200–1500 Hz. The samples of the bubble wraps were cut according to the cross-section area of the impedance tube through a circular die in order to provide uniform contact between its perimeter and the walls of the tube in order to prevent the loss of sound energy on the lateral surface [27]. Three independently prepared specimens were tested for each experimental setup. For each specimen, ten measurements were taken with repositioning after each measurement, thus giving a total of 30 acoustic measurements for each experimental setup. Ten measurements taken from each specimen were considered technical replicates that helped control the variation due to measurement and repositioning. First, the mean sound absorption coefficient was calculated from the ten replicated measurements of each specimen. Then, the result for the entire setup was obtained by averaging the mean values of the three specimens. Hence, three independently prepared specimens can be taken as independent experimental units, while ten measurements from each specimen constitute technical replication.

**Figure 3:**
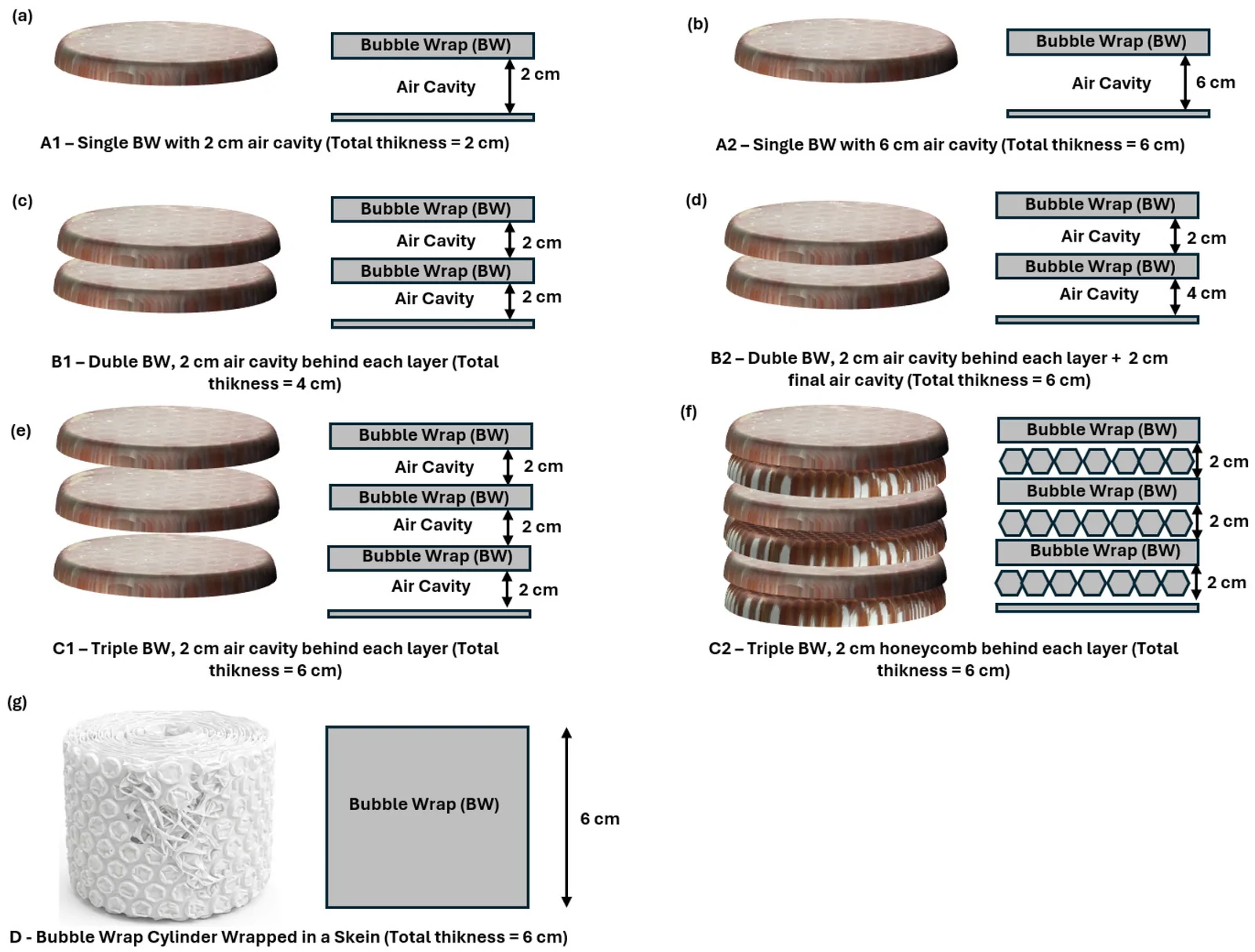
Schematic representation of the seven bubble wrap sample configurations investigated for the acoustic characterization: (**a**) A1—single bubble wrap membrane backed by a 2 cm air cavity (overall sample thickness = 2 cm); (**b**) A2—single bubble wrap membrane backed by a 6 cm air cavity (overall sample thickness = 6 cm); (**c**) B1—double bubble wrap membranes, each followed by a 2 cm air cavity (overall sample thickness = 4 cm); (**d**) B2—double bubble wrap membranes, each backed by a 2 cm air cavity, with an additional 2 cm terminal air cavity (overall sample thickness = 6 cm); (**e**) C1—triple bubble wrap membranes, each backed by a 2 cm air cavity (overall sample thickness = 6 cm); (**f**) C2—triple bubble wrap membranes, each backed by a 2 cm honeycomb core instead of an air cavity (overall sample thickness = 6 cm); and (**g**) D—bubble wrap arranged in a rolled (coil-like) configuration, forming multiple curved layers and enclosed air spaces (overall sample thickness = 6 cm).

The experimental configurations investigated in this study are as follows (Figure 3):

• Configuration A1—single layer of bubble wrap with a 2 cm-thick cavity (overall sample thickness = 2 cm);
• Configuration A2—single layer of bubble wrap with a 6 cm-thick cavity (overall sample thickness = 6 cm);
• Configuration B1—double layer of bubble wrap, with a 2 cm-thick cavity behind each layer (overall sample thickness = 4 cm);
• Configuration B2—double layer of bubble wrap, with a 2 cm-thick cavity behind each layer and an additional 2 cm-thick cavity at the end (overall sample thickness = 6 cm);
• Configuration C1—triple layer of bubble wrap with a 2 cm-thick cavity behind each layer (overall sample thickness = 6 cm);
• Configuration C2—triple layer of bubble wrap, with a 2 cm-thick honeycomb structure behind each layer (overall sample thickness = 6 cm);
• Configuration D—bubble wrap skein structure (overall thickness of the sample = 6 cm).

The systematic variation in the number of layers and of the air gap behind the material allows us to investigate the influence of these parameters on the sound-absorbing properties, similarly to what has been proposed in the literature for other reused materials. The sound absorption coefficient α is calculated from the complex transfer function H_12_ measured between the two microphones, according to the procedure described in ISO 10534-2:2023. The complex reflection factor R is determined as:

(4) $$R=\frac{H_{12}-e^{-\mathrm{jks}}}{e^{\mathrm{jks}}-H_{12}}\cdot e^{2\mathrm{jk}\left(l+s\right)},$$

In Formula (4), k is the wavenumber, s is the distance between the two microphones, and l is the distance from the microphone closest to the sample.

The normal incidence sound absorption coefficient is finally obtained as:

(5) α = 1 − |R|^2^,

In Equation (5):

• α represents the absorption coefficient at frequency (f);
• R represents the complex reflection coefficient.

The results were averaged over 10 measurements for each configuration.

Measurement uncertainty was estimated according to the ISO/IEC Guide 98-3 (GUM) (ISO, 2008) [28], considering the main sources of error: sample positioning, microphone frequency response, and ambient background noise. Three independent specimens per experimental setting were measured, and each specimen was measured ten times with positioning before the next measurement. The average of the ten repeated measurements was determined first for each specimen, then the average of the three independent specimens was taken for each setting. For each frequency point, the standard deviation based on the average value of the three independent specimens was taken as the expression of experimental variability. This method takes into account not only measurement repeatability but also specimen variability without considering repeated measurements as independent specimens. Microphone calibration, temperature, and relative humidity were controlled during measurements in accordance with the experimental procedure. Therefore, these sources of uncertainty were treated as controlled and not as independent statistical uncertainties. Frequency-dependent standard deviations for all settings are given in the Supplementary Materials.

In order to ensure reproducibility among different tested configurations and multiple repetitions, the specimens were mounted using a standard method. Bubbles were always oriented in the same way relative to the incoming sound field. There was no membrane pretension applied, and the bubble wrap was mounted in its relaxed state. The specimen edges were constrained by the cylindrical sample holder, and there were no additional mechanical strains or artificial edge sealing used. In case of multilayer configurations, the layer spacing was set by the cylindrical PVC base, resulting in the 13 mm cavity between the bubble-wrap layers. In case of the honeycomb configuration, the 13 mm cavity was filled by the honeycomb structure. The honeycomb material had a 13 mm thickness, 4 mm cell size, and it was always mounted in the same way for all relevant specimens. In the case of the rolled configuration, the specimen diameter and the number of wraps were always the same for all specimens. The material was mounted without any intentional mechanical compression in order to maintain the packing state without air cell deformation. The same mounting method was applied to all repeated measurements.

In the present work, the experimental configurations have been chosen in such a way as to study the acoustic performance of bubble wrap for various practical installation conditions, rather than forming a full factorial design where all parameters are varied independently of each other. It means that some configurations include variation in several parameters at once, including the number of layers, cavity layout, internal structure, and quantity of material. Whenever possible, differences in two configurations that vary in one parameter only have been used to analyze the influence of the given parameter.

### 2.3. Data-Driven Approach for Predicting the Sound Absorption Coefficient

The prediction of the sound absorption coefficient (SAC) of metamaterials is a demanding task because of the high-frequency dependence of propagation, dissipation, and resonance effects typical for such systems. For multilayered structures made of membranes, honeycomb cavities, and bubble wrap materials, the acoustic behavior is determined by the nonlinear interplay of geometric parameters, medium characteristics, and energy loss mechanisms, which usually makes it challenging to create representative analytical models. Historically, the problem of acoustic performance prediction has been solved using theoretical models based on the wave equation, transfer matrix method, or numerical simulation by the finite element method [29,30,31,32,33]. However, these methods presuppose a deep understanding of the physical parameters of the system and involve high computational costs in the case of heterogeneous configurations and unusual materials. To deal with the problem, this research used a data-driven approach based on machine learning techniques [34,35,36,37,38,39]. Such an approach enables learning the relationship between the geometrical parameters of the metamaterial and its sound absorption coefficient through the analysis of experimental data.

In order to increase the prediction ability of the developed model and guarantee the physical coherence of the obtained results, an additional term based on physical regularization was introduced into the training algorithm of the neural network [40]. In such a way, physical knowledge about the acoustic properties of metamaterials is introduced into the objective function of the network. Contrary to conventional neural networks, which can only predict the statistical relationships between input and output parameters, the Physics-Informed Conditional Neural Network (PICNN) takes into account the physical constraints based on the laws or properties of the studied system [41]. In our research, the physical constraint was not introduced via full-wave equations or the solution of the Helmholtz equation, as the used dataset does not contain any information on the pressure distribution and particle velocity [42]. Consequently, a phenomenological formulation was adopted that exploits a fundamental characteristic of the acoustic behavior of porous materials and membrane metamaterials: the frequency response of the absorption coefficient tends to vary continuously and regularly, showing significant changes only near the resonance frequencies [43].

From a physical point of view, the sound absorption coefficient is defined as in Equation (5).

Since the reflection coefficient depends on the surface acoustic impedance of the material, and this impedance evolves in a generally regular manner across the frequency domain, the absorption coefficient is also a continuous and limitedly oscillatory function [44]. To incorporate this physical information into the neural network, a spectral regularity constraint based on the local curvature of the predicted response was introduced. The monitored quantity was the second derivative of the absorption coefficient with respect to frequency:

(6) $$\frac{\partial^{2}\alpha }{\partial f^{2}},$$

This quantity represents a measure of the local variation in the slope of the absorption curve [45]. High values of the second derivative indicate very rapid changes in the response and may correspond to:

• Numerical oscillations generated by the learning algorithm;
• Overfitting;
• Unphysically unrealistic behavior.

Conversely, moderate values of the second derivative are consistent with the dispersive behavior typical of resonant systems. Since the model is trained on discrete data, the second derivative was approximated using a central finite difference scheme:

(7) $$\frac{\partial^{2}\alpha }{\partial f^{2}}=\frac{\alpha_{i+1}-2\alpha_{i}+\alpha_{i-1}}{\Delta f^{2}}$$

In Equation (7)

• α_i_ is the expected value at the frequency f_i_;
• α_i−1_ and α_i+1_ represent the predictions at adjacent points.

This discrete operator provides a numerical estimate of the curvature of the predicted response. The physical loss term was subsequently defined as the mean square of the curvatures:

(8) $$L_{\mathrm{phys}}\frac{1}{N}\sum_{i=2}^{N-1}{\left(\Delta^{2}\mathrm{SA}C_{i}\right)}^{2},$$

where N is the total number of samples employed in the learning process. As mentioned before, the L_phys_ loss is small if the estimated output varies smoothly over the frequency range and becomes larger if the output of the proposed network shows local oscillations which cannot be attributed to physical phenomena of the system. In terms of the interpretation of the proposed method, this loss function implies a physically driven regularization which smooths the response curve without eliminating the actual resonances but suppressing the artificial fluctuations caused by the neural network [46]. The application of this constraint is quite suitable for the investigated metamaterials, which comprise membrane-, honeycomb-, and bubble wrap-based configurations. This is because:

• Frequency-dependent response;
• Presence of localized resonant phenomena;
• Continuity of the absorption function;
• Limited presence of spectral discontinuities.

To prevent the physical constraint from overpowering the contribution of the experimental data, the regularization term was weighted using a control coefficient (λ_phys_).

The overall objective function was then defined as:

(9) **L_tot_** = L_data_ + λ_phys_L_phys_,

In Equation (9)

• L_data_ represents the mean square error between measured and predicted values;
• L_phys_ represents the term of physical regularization.

This formulation allows for obtaining a model that learns directly from experimental data while maintaining a behavior compatible with the physical principles that govern acoustic absorption in multilayer metamaterials based on bubble wrap.

## 3. Results and Discussion

In this part of the research, the experimental measurements of SAC are done for seven different configurations of bubble wrap to test the effect of configuration, air cavity thickness, and geometry on acoustic performance. These include single-layer samples with variable cavity thickness (A1, A2), multi-layer samples with several cavities (B1, B2, C1), honeycomb design in place of air cavities (C2), and a randomly organized structure (D). Comparison of these configurations would enable the physical mechanisms affecting the acoustic performance (impedance matching, reflection, viscous and thermal losses in the air layer) to be studied along with the effects of structural complexity. Specifically, the effect of the thickness of the sample and frequency-specific absorption is investigated to find out the optimal configurations maximizing performance in low- and mid-frequencies at minimum usage of material. Numerical modeling of these samples provides validation of the model and further understanding of the acoustic properties of bubble wrap as an inexpensive and eco-friendly acoustic solution for residential buildings.

### 3.1. Experimental Sound Absorption Performance

The sound absorption performance of the seven bubble wrap configurations was experimentally evaluated using the impedance tube method. The measured sound absorption coefficient (SAC) curves are presented and compared to investigate the influence of layer arrangement, cavity depth, and structural configuration on the frequency-dependent acoustic behavior of the proposed sustainable insulation systems.

#### 3.1.1. Effect of the Cavity Behind—Single Layer as Membrane (A1 vs. A2)

**Figure 4:**
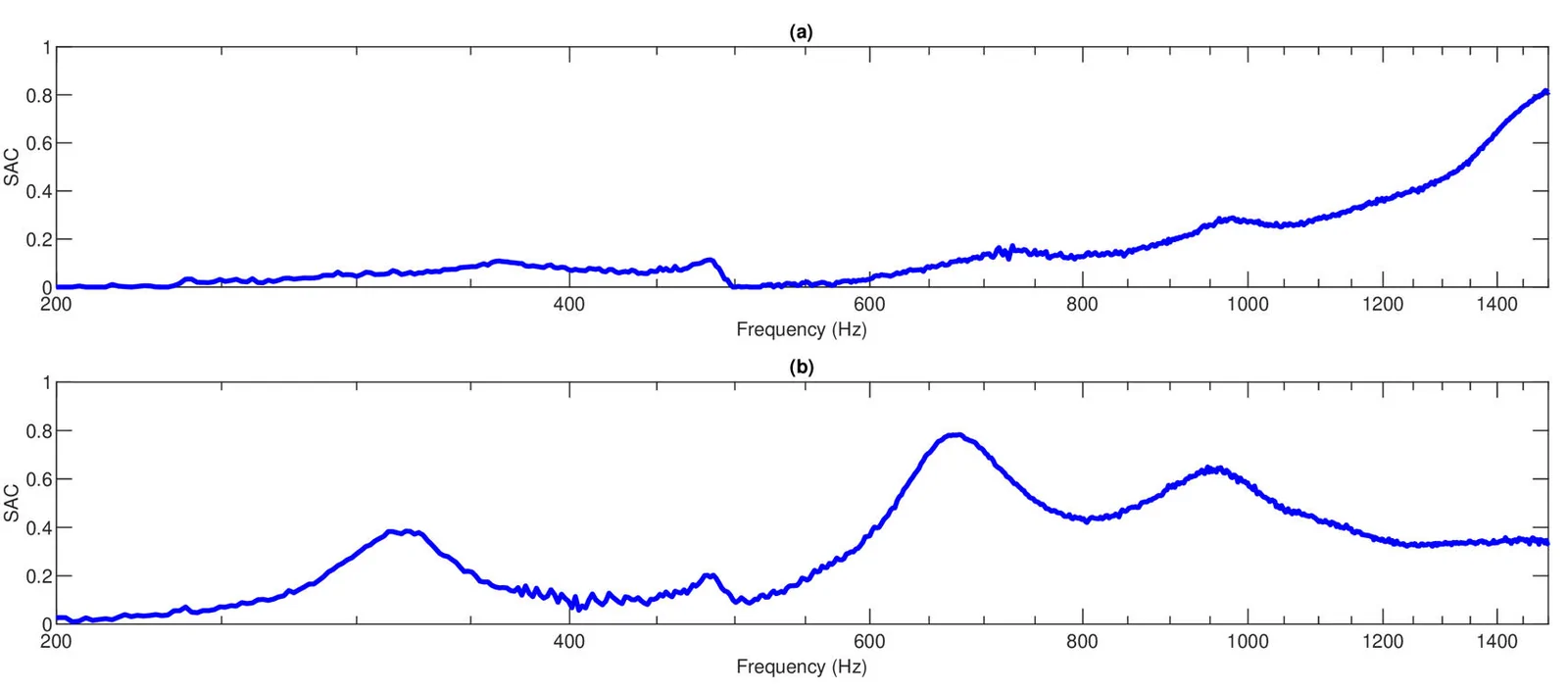
Normal incidence sound absorption coefficient (SAC) measured via impedance tube (ISO 10534-2:2023) for a single layer of reused bubble wrap mounted as a membrane on a rigid backing, as a function of frequency (200–1500 Hz): (**a**) A1 configuration with 2 cm air cavity; (**b**) A2 configuration with 6 cm air cavity.

The analysis of SAC versus frequency dependence for configurations A1 and A2 indicates the nature of the flexible membrane-type resonant absorber associated with an air cavity backed with a rigid wall (Figure 4).

When increasing the air cavity thickness from 2 cm to 6 cm, there is a considerable change in system performance. It results in a decrease in the frequency at which maximum absorption is obtained, but configuration A2 shows multi-frequency absorption. Configuration A1 (d = 2 cm) does not change much in its absorber curve before 800 Hz, after which it increases steadily until 1000 Hz, reaching the maximum level of about 0.82 around 1500 Hz. This can be attributed to the resonance effect at high frequency because of the increased stiffness of the shallow air cavity, resulting in less compliance and a higher resonant frequency. Configuration A2 (d = 6 cm) operates quite differently, with a red shift and three absorption peaks: the first one is located at ~310 Hz (SAC ≈ 0.38), the main one is at 680 Hz (SAC ≈ 0.78), and the second peak is at 960 Hz (SAC ≈ 0.65). This phenomenon can be easily explained through a mass-spring model, where the membrane represents mass and back-cavity air acts like the spring, and the resonance frequency depends on surface mass m:

(10) $$f_{0}=\frac{1}{2\pi }\sqrt{\frac{\rho_{0}c_{0}^{2}}{m\;d}},$$

where ρ_0_ is the air density, c_0_ is the speed of sound, and d is the cavity depth. From this formula, it is obvious that, given a constant membrane surface mass, the frequency decreases as the cavity depth increases in proportion to d^−1/2^.

In passing from A1 to A2, there is an increase in the cavity depth from 2 cm to 6 cm, that is, by a factor of three. In this case, the compliance of the cavity would be increased, and the acoustic stiffness decreased accordingly. As the equivalent air cavity is made to become more compliant, the resonance frequency of the membrane-cavity system should decrease. In a mass-spring approximation, the resonance frequency is related approximately as ($f_{r}\propto 1/\sqrt{L}$). Thus, the increase in cavity depth by a factor of three implies that there is a decrease in the resonance frequency by a factor of ($\sqrt{3}$) and this corresponds to 57.7% of the original frequency. This can explain the trend from A1 to A2, although the multiple resonant peaks for A2 suggest a more complicated acoustic response.

Sound absorption in bubble wrap arises from membrane oscillation, air compression, and viscoelastic and thermal losses. Acoustic pressure difference between the membranes causes vibrations that become more amplified at resonance, leading to a significant conversion of acoustic energy into mechanical vibrations. Some part of this acoustic energy is dissipated because of mechanical and viscoelastic losses, as well as because of the repeated deformation of the membranes and air bubbles, causing hysteresis and the conversion of mechanical energy into heat [47].

There is also an additional loss associated with thermoviscous effects in the rear cavity, when the membrane vibrates, compressing and rarefying the air, causing the thermoviscous dissipation close to solid boundaries and the conversion of acoustic energy into heat. In other words, the efficiency of absorbers is determined by the interaction of the inertia of the membrane, air compliance, and all these losses combined. There is no way to explain the three absorption peaks of A2 (310, 680, and 960 Hz) using only the mass-spring resonance. This may be explained by the coupling of the resonance of the membrane as a whole and higher-order modes of oscillation of the membrane, together with local dynamic properties of individual bubble cells—namely, the cellular nature of the material allows for the creation of many pneumatic/mechanical elements that create coupled resonances [48]. Hence, increasing the cavity depth from 2 cm to 6 cm has two consequences: reducing the frequency to promote low-frequency absorption and changing the coupling conditions of the membrane cavity to create multiband behavior. A2 is distinguished not only for having a high value of SAC (≈0.78), but also for maintaining strong absorption in the range of 600–1000 Hz.

#### 3.1.2. Effect of the Cavity Behind—Double Layer as Membrane (B1 vs. B2)

**Figure 5:**
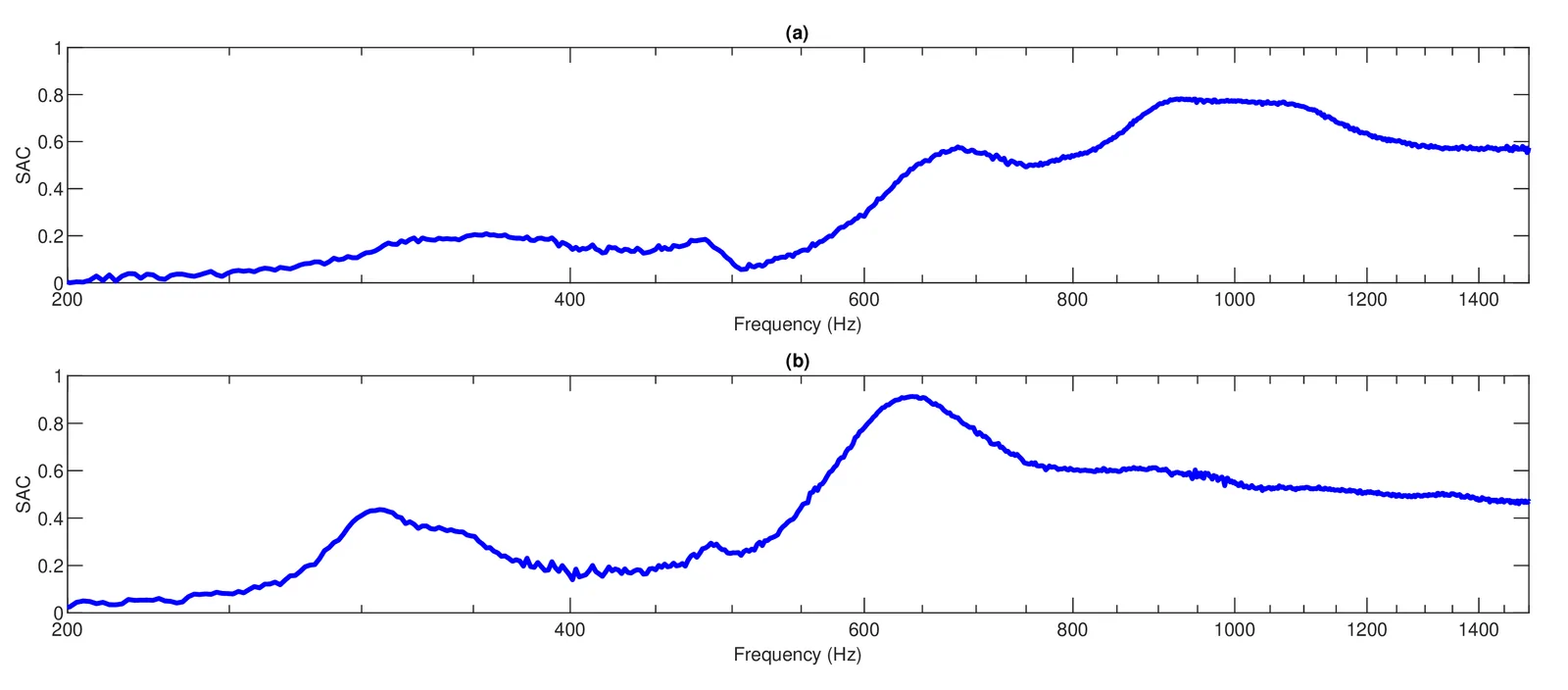
Acoustic absorption coefficient (AAC) at normal incidence determined by means of an impedance tube (ISO 10534-2:2023) for two-layer reused bubble wrap placed as a membrane on a rigid substrate as a function of frequency (200–1500 Hz): (**a**) B1 construction with 2 cm air cavity per layer (total cavity depth: 4 cm); (**b**) B2 construction with additional 2 cm air cavity beyond the second layer (total cavity depth: 6 cm).

Configurations B1 and B2 are characterized by much better sound absorption properties compared to A1 and A2 (Figure 5). The enhancement is due not to the larger quantity of materials but to the layering and division of the air volume into coupled cavities. The configuration B1 (two layers, each 2 cm cavities) widens the absorption range to include medium frequencies, compared to single-layer A1. The peak in absorption occurs at ~660 Hz (SAC ≈ 0.58) and is followed by high values of absorption (SAC ≈ 0.78) in the range 850–1100 Hz. In the case of B2, where the thickness of the structure was increased and three cavities were used (each 2 cm, total 6 cm), even stronger effects were noticed. Namely, there was an absorption peak at ~280 Hz (SAC ≈ 0.43) and a clear resonance at 640 Hz (SAC > 0.91—the maximum value). Above 800 Hz, absorption remains substantial (0.50–0.60).

By considering B1 and B2, it is clear that not only does the thickness increase result in shifting the response spectrum to low frequencies, but it also causes changes in the absorption spectrum, resulting in the appearance of several resonant zones. The above-mentioned advantages can be attributed to the multi-degree-of-freedom mass-spring-damper system where each membrane and cavity serve as a coupled element. While the one-layer structure works as a simple mass-spring system, adding one more membrane brings about the dynamic coupling of several resonating elements. Each bubble-wrap layer serves as a surface-mass element with its own elastic and dissipative properties, and the cavities work as compliance elements. This results in the appearance of coupled resonances, splitting of natural frequencies, and surface impedance changes, which allows obtaining high absorption coefficients and increased bandwidth [49]. Another factor that should be considered is reflections taking place within the multilayered structure. Waves are both reflected and transmitted by the membranes and cavities. Thus, the path of the wave increases due to additional reflections. It increases the probability of energy conversion into structural degrees of freedom and thus dissipation. Concerning impedance matching, the layering changes the surface impedance; its resistive component is related to system losses, and the reactive component is related to the mass of the membrane and cavity compliance. The absorption is maximized when the ratio of these components is close to air impedance. Quantitatively, the presence of the second layer provides degrees of freedom allowing independent variation in the reactive and dissipative components, which reduces the reflection from the surface.

The fact that SAC is larger than 0.91 at ~640 Hz for B2 proves the efficacy of the multilayer configuration, although the lack of surface impedance measurements making sure that Zs/Z0 ≈ 1 does not allow calling this critical absorption but favorable impedance matching. The comparison of B1 (two 2 cm cavities) with B2 (additional 2 cm cavity, total 6 cm) shows the influence of the additional air volume. For the mass-spring system, the natural frequency decreases approximately as d^−1/2^, as in B2 there is an absorption peak at 280 Hz missing in B1. The complex behavior at 640 Hz, at which the value of SAC is greater than 0.91, arises due to the interaction between the two membranes and their cavities. The performance improvement of B1 and B2 results from dissipation by both mechanisms. Having two polymer membranes increases flexible surfaces, which allow for improved dissipation by means of vibration and viscoelastic hysteresis when excited harmonically. In the case of bubble wrap, the presence of segmented air cells complicates the process: bubble deflection brings in the elastic part and increases dissipation as a result of interaction between the membrane dynamics and compressed air. Moreover, intermembrane air cavities cause thermo-viscous losses caused by interaction of oscillating air with solid surfaces and arising velocity and temperature gradients during the compression–expansion cycle.

On the whole, the use of stacked bubble wrap is a good way to improve and tune sound absorption. Moving from one-layer A1 to B1 and B2 not only leads to increasing SAC peaks but changes spectrum distribution: B1 provides improvement in the middle frequency range and B2—efficient absorption of low frequencies (SAC > 0.90 at 640 Hz) together with reliable high-frequency absorption. This proves that cavity depth, cavity location, and number of membranes are vital parameters in the design process, and adjusting them independently will allow us to create multilayered systems tailored for optimal energy absorption, broadband operation, and low-frequency response capabilities—a bubble wrap-based resonant acoustic system.

#### 3.1.3. Effect of Honeycomb Structure in the Cavity—Triple Layer as Membrane (C1 vs. C2)

**Figure 6:**
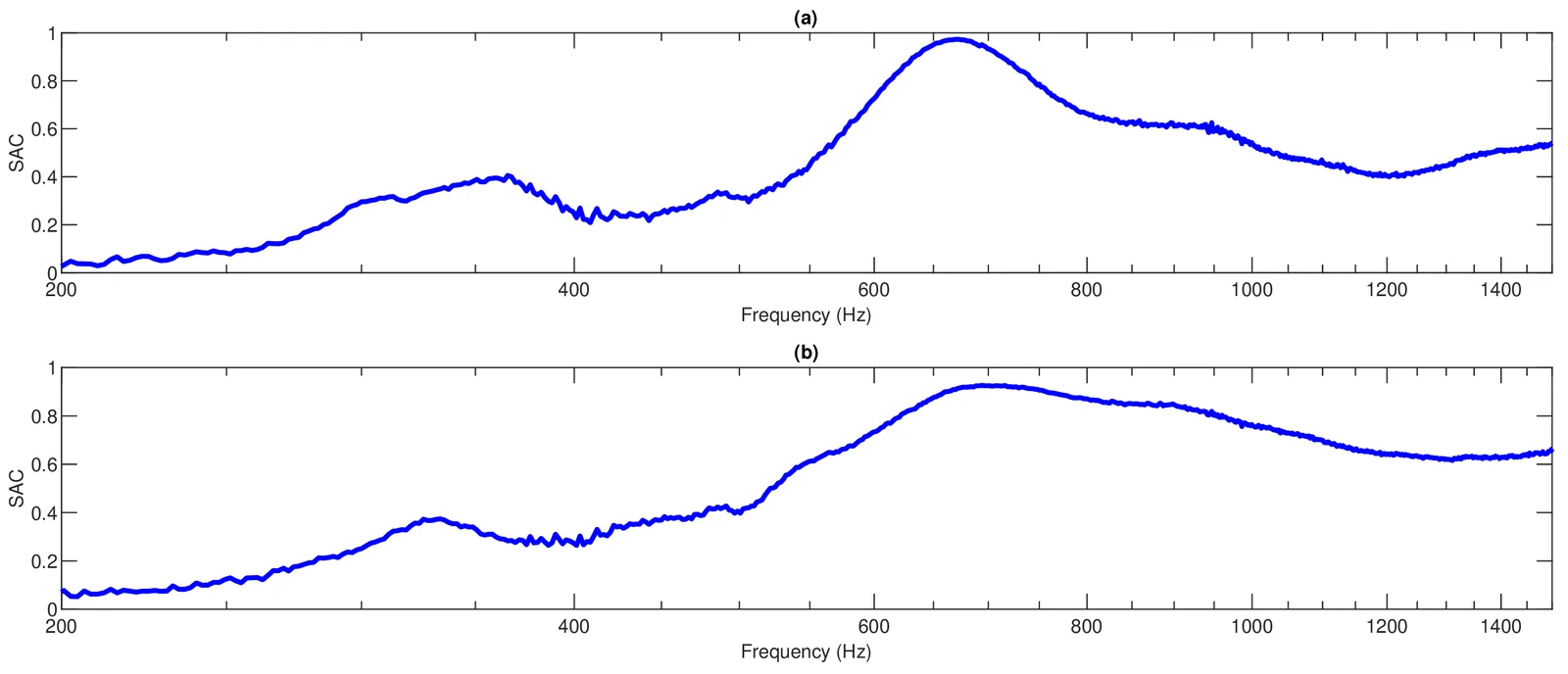
SAC at normal incidence obtained by the impedance tube method (ISO 10534-2:2023) of three layers of bubble wrap that has been reused and used as a membrane with rigid backing as a function of frequency (200–1500 Hz): (**a**) C1 configuration: 2 cm free air cavity between layers; (**b**) C2 configuration: honeycomb structure in cavity between layers.

The SAC analysis of three-layer structures indicates another improvement—multilayer design controlling not only the magnitude of absorption but also its frequency spectrum shape. Comparing open-cavity design C1 to honeycomb core design C2 stresses the significance of cavity geometrical parameters (Figure 6). Design C1 (three layers, ~2 cm cavities) demonstrates an obvious resonance effect—a low-frequency peak of ≈360 Hz (SAC ≈ 0.40) and a near-unity peak of ≈0.97 at 660 Hz, showing high energy dissipation in resonance. Nevertheless, this peak is narrow—absorption is decreasing starting from 750 Hz and becoming stabilized at 0.45–0.50 at 1000–1400 Hz. Honeycomb core design C2 has similar characteristics at low frequencies (peak SAC ≈ 0.38 at 320–340 Hz) but behaves differently at mid-to-high frequencies—a peak of ≈0.93 while keeping SAC above 0.80 within a wide range 620–950 Hz, and stable performance (SAC ≈ 0.65) above 1000 Hz [50].

The configuration C1 may be characterized using the approach where three membranes and cavities constitute the multi-degree-of-freedom system, the three membranes acting as masses and dissipative elements, and air layers functioning as compliant elements, thus leading to the occurrence of several resonant frequencies and the change in impedance of the surface. At the frequency of 660 Hz, the dynamics of the membranes and cavities become highly effective for dissipation of the sound energy, leading to an almost unitary value of SAC due to the correct interrelation between the reactive and dissipative components of the impedance. However, the deviation from the impedance of the air takes place at the resonances, leading to a considerable increase in reflection and decrease in the value of SAC from 750 Hz [51].

Application of honeycomb geometry leads to a relatively modest peak value decrease (from around 0.97 to 0.93), yet a significant bandwidth increase (which makes acoustic behavior less selective and more broadband). Honeycomb geometry of C2 affects the acoustic performance of the cavity because it increases the ratio of solid-to-air surface and restricts motion of air in the cavity, thus increasing the interaction between waves and walls. Two main mechanisms can be considered as plausible causes of the effect in question. First, it is viscous losses due to the formation of velocity gradients at channel boundaries and dissipation of part of the kinetic energy in the form of heat. Second, thermoacoustic losses due to heat transfer between the solid and air can also be considered as a possible reason for the observed effect. Both effects are supposed to depend on cell geometry, frequency, and air properties. However, airflow resistivity and surface impedance of the honeycomb core have not been measured in the current study; thus, both mechanisms cannot be independently assessed as dominating ones. It should be noted that the presented interpretation is based on the physically plausible description of acoustic behavior.

As a result, the more general response may have more dissipative properties that lower the Q-factor of the resonant peak. In fact, as demonstrated by the reduction in the value of SAC from ~0.97 to ~0.93, this is a trade-off between decreased peak efficiency and significantly enhanced bandwidth: C2 demonstrates SAC > 0.80 in the range of ~620–950 Hz and SAC > 0.65 for frequencies higher than 1000 Hz, showing that the effect of the honeycomb structure extends far outside the resonant zone. Efficiency can be described as a result of dissipative processes on different levels. On a macro-level, the three oscillating membranes can dissipate energy due to viscoelastic deformation, while, on the micro-level, each bubble cell adds additional elasticity and damping to the structure. Moreover, the honeycomb structure can provide another fluid dynamic dissipation process based on the air flow inside the narrow channels of the structure. Since all these processes were not separately investigated, they should be regarded as possible explanations for the observed acoustic characteristics.

The comparison of C1 and C2 shows the efficiency of the honeycomb configuration for the conversion from a resonant to broad-band absorber. Although C1 achieves the greatest peak value (SAC ≈ 0.97 for f = 660 Hz), it still has a narrow bandwidth. On the other hand, although C2 produces a slightly lower peak value (SAC ≈ 0.93), it maintains SAC ≈ 0.80 for ~330 Hz and SAC ≈ 0.65 at frequencies above 1000 Hz. The comparison indicates that the effectiveness of the absorber must be considered not only by the peak value of SAC but also by the bandwidth of the absorption. C2 thus can be regarded as a multi-scale system, where resonance, impedance matching, and dissipation effect could jointly work.

#### 3.1.4. Volumetric Mass Configuration—Bubble Wrap Wrapped in Skein (D)

**Figure 7:**
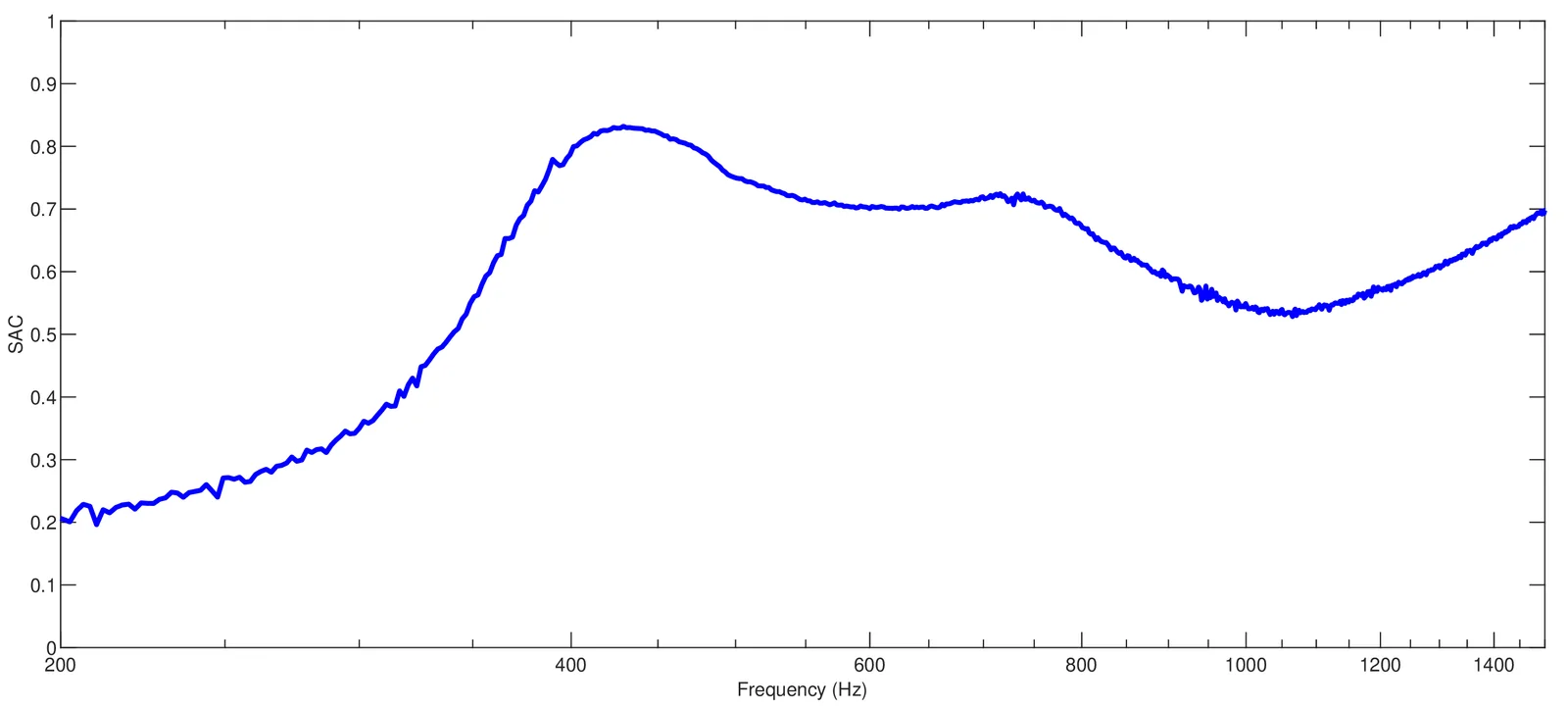
Reused bubble wrap Sound Absorption Coefficient (SAC) under normal incidence conditions by means of impedance tube measurement (ISO 10534-2:2023) for reused bubble wrap in coil (ball of yarn like) form, with total thickness of 6 cm as a function of frequency (200–1500 Hz).

Rolled/compressed bubble wrap (6 cm overall thickness) is entirely different from the layered membrane structures. Compression causes the formation of a 3D heterogeneous structure of the polymer matrix, air bubbles, and air pockets, thus providing for broadband dissipation rather than a single resonance, with both local resonances and dissipation inside the bulk material being possible (Figure 7). The experimental results prove that sound absorption coefficients become apparent at frequencies starting from 300 Hz (SAC = 0.35) to 440 Hz when SAC reaches the maximum (≈0.83). An increase in frequency leads to a constant SAC of more than 0.70 in the frequency range of 500–800 Hz that can be considered as the effective absorption band. Then, SAC decreases slightly to 0.53 at 1000 Hz to rise to 0.70 at 1500 Hz. The absence of sharp minima and high SAC in a wide frequency range shows that such a structure does not operate in a manner of a discrete cavity resonance system. This may be explained by the fact that in the rolled structure the role of the heterogeneous acoustically dissipative medium is played by the sound wave interaction with the complex system of air cavities and polymeric matter [52].

Air channels exist in the form of interspaces between layered stacks and bubbles. The key characteristics of these channels are small areas/volumes, tortuosity, and non-uniform sizes. When air interacts with the polymer surface during excitation, it has a higher area-to-volume ratio in comparison with the open-air cavity, and this might lead to higher energy dissipation at the surface. One of the possible ways of energy dissipation is viscous dissipation from oscillations of air in small interspaces. No-slip conditions on polymer surfaces can create velocity gradients in the acoustic boundary layer that may dissipate kinetic energy as heat.

The second potential loss source involves thermal interaction between the oscillating air and the polymer walls. In this regard, it is necessary to note that the vibration/contraction process within microcavities can lead to temperature changes and transformation of a part of acoustic energy into thermal energy. Thus, viscous and thermoacoustic losses can indeed influence (at least in a qualitative way) the high absorption observed outside of the resonance peak around 440 Hz. At the same time, it is necessary to note that the resistivity of the airflow and surface impedance of the rolled shape were not investigated in the current study. Consequently, these loss sources cannot be separately quantified or identified as dominating ones. However, the system is characterized by some resonant elements, namely, first, separate air bubbles surrounded by polymer membranes can be considered dynamically because of the deformation of these membranes and the compression of the air inside them; second, air bubbles located in between the rolled layers represent small acoustic cavities of irregular shape. In addition, inhomogeneous pressure and deformations are generated by the rolling of layers, which means that the mass-spring system becomes inhomogeneous rather than homogeneous [53].

The additional absorption could be caused by the very characteristics of the polymer material: the energy related to the deformation of the bubbles’ walls and layers could be partially converted to the thermal form due to the effect of the hysteresis and viscosity of the polymer. Due to the fact that the folded material contains more interfaces and dissipative elements than a one-dimensional film, the structural dissipation exists in the whole thickness of 6 cm and might be responsible for the off-resonant absorption. It should be emphasized that both viscoelastic dissipation and thermo-viscous effects of the air might be responsible for the broadband absorption phenomenon. Furthermore, the complex structure with high dissipation might perform better impedance matching than a rigid surface and a large amount of air. Consequently, the acoustic wave would enter the material and meet many interfaces until being reflected. In the vicinity of 440 Hz, the resistance and reactance of the impedance of the material might be optimally organized in order to provide the penetration of the acoustic wave inside the material and its dissipation in solid and gas media.

In general, the structure being discussed cannot be regarded as either just a resonant structure or simply a dissipative structure, but rather as a multiscale structure where the contributions of both resonating and dissipating phenomena can be involved. It is really impressive how well the rolled structure performs in terms of absorption: SAC ≈ 0.83 at 440 Hz, SAC > 0.70 for 500–800 Hz, SAC ≈ 0.70 at 1500 Hz—a broadband behavior that cannot be caused by a resonant phenomenon only. If compared with layered structures having cavities, the rolling technique alters the behavior of the system from the resonant-cavity one to the broadband dissipative one. Micro-cavities filled with air, tortuous channels, viscoelastic behavior of the polymer material, and the thermo-viscous effects in the air can all be responsible for dissipation over the broader range of frequencies. While the structures with the cavities allow tuning to some resonant frequencies, the rolled/bulk structure allows having broadband absorption behavior.

The problem associated with the current experiment is that the present experimental design lacks a complete factorial design. In some cases, all four variables change simultaneously, such as the number of layers, cavity division, inner structure, and material amount. As a result, it is impossible to distinguish the individual contribution of each variable based on the existing set of data, and there will always be overlapping between the geometrical and material variables. Although comparisons made in this experiment can give an idea about the acoustical characteristics of a particular configuration, they cannot give a statistical evaluation of the contribution of each individual variable. Thus, to get numerical data concerning the contribution of each variable, a complete factorial or response surface experiment needs to be conducted.

#### 3.1.5. General Comparison of Optimal Configurations

**Figure 8:**
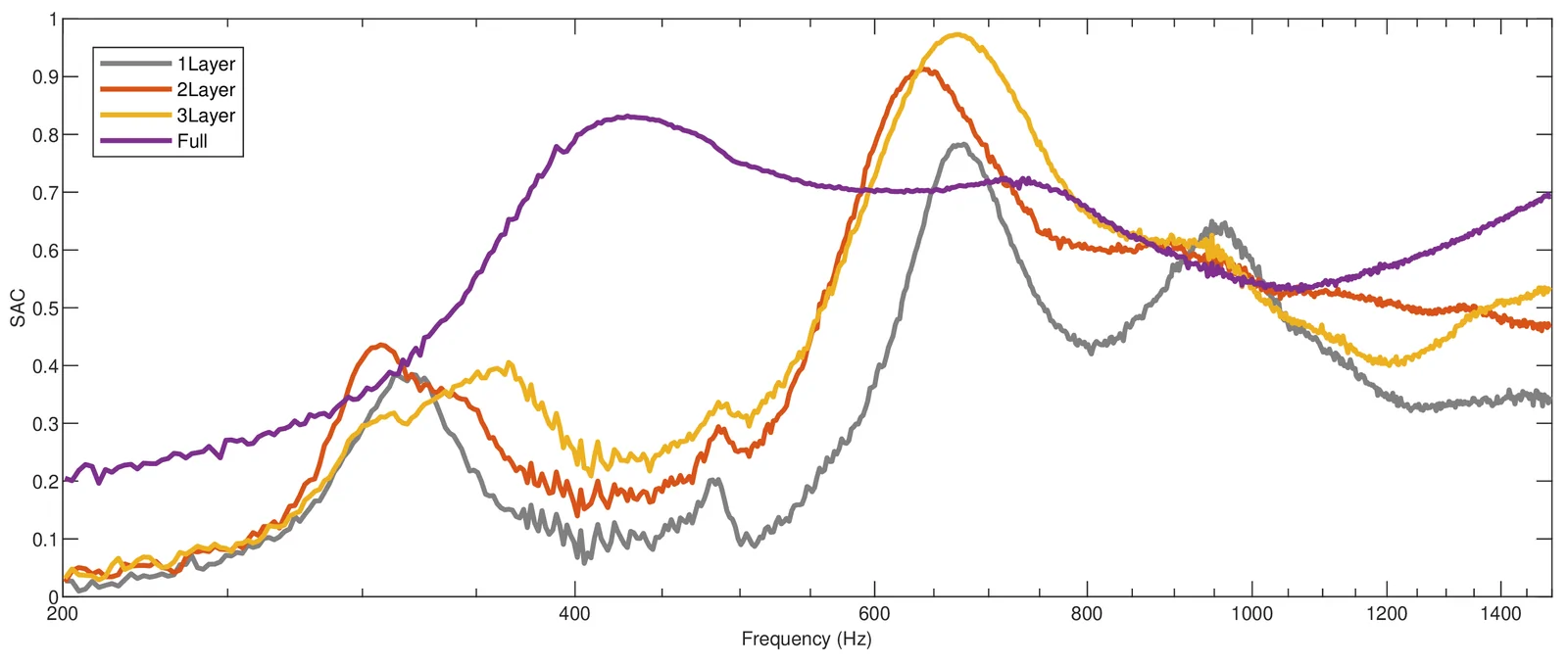
Comparison of the normal incidence sound absorption coefficient (SAC) measured via impedance tube (ISO 10534-2:2023) for the four most representative configurations of reused bubble wrap, all with a total equivalent depth of 6 cm, as a function of frequency (200–1500 Hz): single layer with 6 cm air cavity (1 Layer, gray, A2); double layer with total 6 cm air cavity (2 Layer, red, B2); triple layer with total 6 cm air cavity (3 Layer, yellow, C1); full rolled mass of 6 cm thickness (Full, purple, D).

Comparison of the four configurations that perform best acoustically shows that there is a definite progression in the acoustic behavior of bubble wrap depending on how it is configured geometrically (Figure 8). While each configuration has a total thickness of 6 cm, differences in layering and volume of air lead to distinctly varied acoustic responses, indicating the shift from resonating and narrow band systems to a system with more broadband absorbers [54]. Configuration A2 (single bubble wrap layer, 6 cm rear cavity) represents the simplest design, exhibiting a single dominant resonance at 680 Hz (SAC ≈ 0.78, Table 2), with sharp absorption decline away from resonance—characteristic of a narrow-band resonant absorber. B2 configuration, introducing another bubble wrap layer which divides the air volume into subdomains, provides substantial efficiency enhancements: the absorption peak gets lowered (680→640 Hz) while SAC rises sharply to 0.91. The new layer introduces an additional degree of freedom, affecting coupling between the acoustic field, vibration of the membrane, and the cavities inside, turning more incident energy into structural vibration, which gets dissipated by viscoelastic and air–structure interactions. C1 configuration (3 layers, 2 cm cavities) achieves even better results: SAC ≈ 0.97 at 660 Hz—the maximal value obtained. An additional layer increases elasticity and the air/material interface, increasing structural–acoustic interaction; the system operates like a mass-spring-damper system, whose interactions change impedance and dissipation. Resonance frequency remains stable within the range 640–680 Hz for all configurations, which means that the addition of layers increases resonance efficiency without significantly moving the characteristic frequency—the specific resonance depends on air compliance, membrane characteristics, and cavity dynamics. In addition, there is a secondary low-frequency resonance (~300–360 Hz, SAC ≈ 0.40) in the C1 configuration, absent in A2, due to increased dynamic degrees of freedom.

Configuration D (Full/rolled) exhibits an entirely different pattern, exhibiting a wider spectrum with its first peak occurring at ~440 Hz (SAC ≈ 0.83) and appearing at much lower frequencies compared to the layered ones. Rolling and packing lead to many micro-cavities and non-linear pathways, thus making this configuration a heterogeneous system rather than separate cavities. The tortuous internal paths make for a longer acoustic path length, leading to viscous and thermo-viscous losses and, together with polymer elasticity, lead to viscoelastic losses—thus resulting in a more homogeneous, resonance-free absorption.

This makes Configuration D a dissipation-based heterogeneous material rather than a normal porous material. Unlike Configurations A2, B2, and C1 that exhibit clear peaks with a fast decrease, Configuration D exhibits SAC ≥ 0.53 between 350 and 1500 Hz, reaching SAC = ~0.70 at 1500 Hz—indicating a clear transition to dissipation-based mechanisms. In general, the four configurations above show two design strategies. Configurations A2, B2, and C1 utilize the membrane resonance and cavity-layer interaction principles, with an increasing peak of SAC of 0.78 to 0.91 to 0.97 due to layer increases, which makes them suited for selective narrow-band absorptions. However, Configuration D trades off the efficiency (0.83 compared to 0.97) in favor of bandwidth, thus making it suitable for broad-band application. In practice, configuration C1 is excellent for selective absorption between 650 and 670 Hz, and configuration D is advantageous for broad-band absorption of low to medium frequencies. From the above, there is clearly a trend of shifting from resonance-based to broad-band absorption in complex geometry designs, and it is clear that the acoustics of bubble wrap depends on its geometry parameters such as number of layers, distribution of air pockets, flexibility, and propagation channels.

In order to obtain a more comprehensive evaluation of the acoustic properties of the investigated bubble wrap configurations, some supplementary parameters apart from the maximal value of the sound absorption coefficient were computed. Table 3 presents the values of the Noise Reduction Coefficient (NRC), Sound Absorption Average (SAA), and the average value of the sound absorption coefficient (SAC) within the range of frequencies 200–1500 Hz. In order to detect possible differences in frequency-dependent characteristics, the average values of SAC were additionally calculated separately for three frequency intervals: 200–500 Hz, 500–1000 Hz, and 1000–1500 Hz. This set of indicators enables the comparative evaluation not only of the maximal absorption coefficient but also of the total absorption properties of configurations along with their frequency dependence. Specifically, the values of SAC per frequency interval give an additional insight into the question of how the acoustical properties of each configuration are localized in the frequency spectrum. The obtained values are presented in Table 3.

The results in Table 3 show a clear improvement in the overall acoustic performance as the bubble wrap configuration becomes more acoustically structured. A1 exhibits the lowest integrated performance, with an NRC of 0.10 and an average SAC of 0.236 over 200–1500 Hz.

The introduction of additional layers and modified cavity configurations progressively increases the integrated indicators. A2, B1, and B2 show intermediate performance, with average SAC values of 0.370, 0.472, and 0.486, respectively. C1 and C2 further increase the overall absorption, reaching average SAC values of 0.496 and 0.623, while their NRC values increase to 0.30 and 0.45. The frequency-band results also reveal that the absorption is not uniformly distributed over the investigated spectrum. For most configurations, the 500–1000 Hz band provides higher average absorption than the 200–500 Hz range, indicating a greater effectiveness in the mid-frequency region. C2 shows particularly high performance in this range, with an average SAC of 0.801. In contrast, D provides the highest low-frequency average SAC (0.545) and the highest overall NRC (0.50) and SAA (0.55), indicating a broader and more balanced absorption response. These results demonstrate that peak SAC alone does not fully describe the acoustic performance of the configurations.

It is worth noting that the current experimental setup aimed at determining the acoustic characteristics of the bubble wrap configurations as a whole, but not at independent measurement of the contribution of each element separately. As a result, no control experiments with an empty cavity, flat LDPE membrane, honeycomb core only, and commercial absorber were carried out. Thus, the sound absorption value cannot be divided into contributions of each element mentioned above. In turn, comparison of the studied configurations demonstrates that there are changes in the acoustic characteristics of the configuration depending on its configuration. Control measurements with the help of individual elements and a commercial absorber need to be conducted.

### 3.2. Physics-Informed Predictive Modeling of Sound Absorption

As a supplementary approach to the aforementioned experiments, a physics-informed predictive model based on a Conditional Neural Network (CNN) was proposed to predict the SAC of the tested configurations of bubble wrap. Contrary to traditional data-driven models that employ the experimental data solely, the proposed approach uses constraints based on the physics of the phenomenon in question that represent the underlying principles governing the mechanism of sound propagation and dissipation [55].

#### 3.2.1. Neural Network Architecture, Training and Hyperparameter Optimization

In order to achieve model reproducibility and estimate the model’s generalization ability in an appropriate way, the network development algorithm has been modified by explicitly splitting the dataset into training (70%), validation (15%), and test (15%) sets. This strategy was employed to avoid performance evaluation of the model on the dataset that has already been used to tune the weights of the network. In order to avoid any information leak between the correlated frequency points of the same experimental acoustic response curve, the database was partitioned at the level of the configurations themselves, and not through random allocation of individual frequency points. As such, the 70/15/15 ratio was implemented on the experimental configurations, which means that 70% of all configurations were allocated to the training set, while 15% of the configurations went to the validation set and the other 15% to the independent test set. All of the frequency points that were part of the same bubble wrap configuration remained in the same subset, without being divided into different subsets. This approach ensures that the test set consists of complete frequency-dependent response curves of experimental configurations that have not been included in the model training process.

The input of the model includes five features that represent the acoustic response of the bubble wrap, while the output of the network is SAC. The five input variables that were utilized by the PICNN include Frequency, Layer, Thickness, HC, and Full. Frequency refers to the frequency of measurement, measured in Hz. Layer refers to the number of layers of bubble wrap used in the configuration, and it is denoted in integers. Thickness refers to the nominal thickness of the bubble wrap material, denoted in mm. HC refers to the thickness of the honeycomb or cavity subdivision, denoted in mm; however, in case of no honeycomb in the configuration, this input variable takes on a value of zero. Full refers to a binary categorical input variable that refers to either the presence (1) or absence (0) of the rolled configuration. Prior to being entered into the neural network model, the numerical input variables were normalized in order to train the model and also ensure comparability in terms of numerical values of the input variables. The categorical variable was binary-encoded.

The neural network is feed-forward and fully connected, having three hidden layers with 64, 64, and 32 neurons, respectively. Hidden layers use hyperbolic tangent (tanh) as an activation function, while the output layer uses only one linearly activated neuron due to the continuous nature of the target variable. Before training, the input features and the SAC were scaled through z-score normalization. To prevent any leakage of information, the parameters of scaling were computed only on the training set and then were applied to the validation and test sets. The same approach was used when transforming the predictions back to their original scale. The dataset was split into a training set for tuning of the weights and separate validation and test sets. Splitting was done using a randomized procedure with a fixed seed of 42 in order to make results reproducible. The validation set was used solely for selection of hyperparameters and observing the training process, while the test set was kept out of the development process entirely.

The objective function of the model consists of two parts—the first one is an error term based on the experimental data and the second one is a regularization term that comes from the expected spectral response:

(11) **L_tot_** = L_data_ + λ_phys_L_phys_,

The first term, Ldata, is given by the squared difference between the calculated SAC and the experimental data SAC. The second term represents the spectral smoothness constraint, which is achieved by penalizing any very fast changes in the curvature of the response with respect to the frequency:

(12) $$L\mathrm{MSE}\left(\frac{\partial^{2}\mathrm{SAC}}{\partial f^{2}},0\right),$$

This quantity is not used as the constitutive law for bubble wrap. It is an additional physics-inspired condition, intended to ensure the consistency of the frequency dependence of the predicted solution and minimize any oscillatory behavior in the predictions not observed experimentally. The relative weighting of the physics-inspired term, λ_phys_, was chosen as part of the validation process among different candidate configurations.

There was no need for an independent boundary condition term, since the construction of the model relied solely on the continuity of the frequency response and on the experimental data. Hence, a λ_bound_ term was not included in the final model.

The hyperparameter tuning was done using only the training and validation data sets (Table 4). Different values were considered for the number of neurons, the number of hidden layers, the learning rate, and the weighting parameter for the physics-informed term.

The weight optimization procedure was carried out using the Adam algorithm, where the learning rate was selected during the hyperparameter optimization stage. The maximum number of epochs and the early stopping conditions were chosen based on the validation performance in order to reduce the probability of overfitting.

Performance was assessed separately for training, validation, and test datasets using the coefficient of determination (R^2^), mean absolute error (MAE), root mean square error (RMSE), and normalized root mean square error (NRMSE). It is important to note that the test dataset stayed fully independent during the entire process of model selection and training. Thus, the performance obtained for the test dataset serves as an estimate of the generalization ability of the neural network concerning observations which were not involved in weight or hyperparameter tuning.

The above approach also makes it possible to separate the issues of fitting and prediction. Hence, the use of R^2^ calculated on the basis of the whole sample as a criterion of generalization of the model can no longer be used. The comparison of the results of training and testing sets will help detect the presence of overfitting.

The presented PICNN is a compact data-driven representation of the acoustic behavior of the considered bubble wrap structures, taking into account the dependency of the sound absorption coefficient on the frequency. The major advantage of the presented PICNN is the ability to take into account both frequency- and configuration-dependent variables for making predictions. Moreover, the physics-informed term imposes the smoothness of the spectrum by the second-order derivative of the predicted SAC with respect to frequency and thus decreases physically unrealistic oscillations in the predicted responses. From the physical point of view, the high level of correlation between the predicted and measured SACs demonstrates that the developed model captures the frequency dependency of SAC regarding the variation in number and thickness of layers and honeycomb configuration. Therefore, the PICNN can be viewed as a surrogate model of the physical process but not the physical model itself.

#### 3.2.2. Qualitative Comparison Between Experimental and Predicted Curves

**Figure 9:**
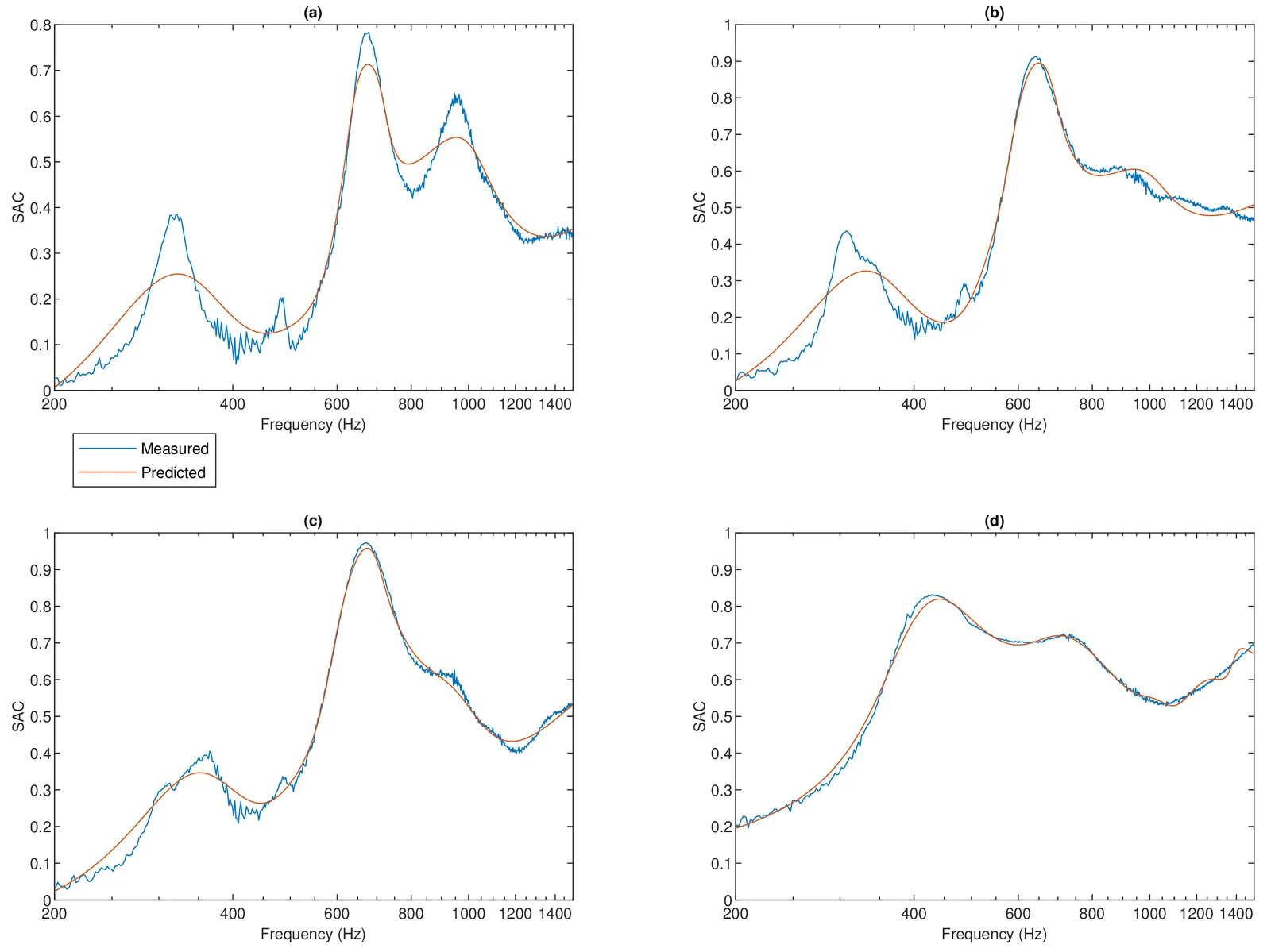
Plot depicting comparison between experimental SAC curve and SAC curve predicted by PICNN of bubble wrap material having total thickness of 6 cm: (**a**) A2—single layer of bubble wrap having 6 cm air layer behind; (**b**) B2—double layer of bubble wrap having 2 cm air layer behind each layer and one extra 2 cm air layer; (**c**) C1—triple layer of bubble wrap having 2 cm air layer behind each layer; (**d**) D—randomly entangled bubble wrap material.

Figure 9 shows the comparison between the experimental SACs and those predicted by the suggested Physics-Informed Conditional Neural Network (PICNN) for four representative bubble wrap samples whose total thickness was 6 cm: A2, B2, C1, and D. The four chosen samples have been used in order to assess the model’s ability to properly simulate the acoustic behavior of multilayer samples with different structural designs.

Figure 9 compares measured and PICNN-predicted SAC curves for four bubble wrap configurations (a–d), each with a constant 6 cm total thickness, across the 200–1500 Hz range most relevant to architectural and building acoustics. Bubble wrap’s acoustic behavior combines cavity-resonator effects (air bubbles enclosed in plastic film) with coupled air-layer dynamics resembling Helmholtz resonators or mass-spring-damper systems, depending on constraint and macroscopic porosity. Configuration A2 (one layer, 6 cm air gap) demonstrates a clear multimodal experimental spectrum: a small peak at around 320 Hz, a large main peak at approximately 660 Hz (SAC ≈ 0.8), and a small secondary peak at about 960 Hz—a typical behavior of a flexible membrane attached to a rigid wall and creating cavity standing-wave resonance with membrane vibration. The PICNN algorithm predicts the general trend of the behavior and finds the 600–1000 Hz absorption maximum, but there are considerable deviations: the 320 Hz peak is not predicted, and the two higher peaks are combined into a single low one (~0.6). It means that there are strong nonlinear or structural couplings of the thin membrane with a large cavity which cannot be completely modeled by the physics-informed part of the algorithm [56].

The second configuration of B2 involves segmentation of the cavity using two layers of bubble wrap to make three 2 cm pieces. The segmentation greatly alters the curve with a peak at 310 Hz (SAC ≈ 0.43), and then the main peak at 640 Hz (SAC > 0.9), followed by a plateau with high absorption (0.5–0.6). Performance of PICNN is greatly enhanced for such cases since it correctly predicts the presence of the low-frequency peak, although it underpredicts the amplitude of the peak (~0.3) and shifts its location a little, and correctly identifies the main peak and further trend up to 1400 Hz [57].

Configuration C1 (three layers, 2 cm intervals) results in increased stratification density, thus restricting air movement and enhancing thermal-viscous damping. In practice, this leads to an intermediary peak at 380 Hz and a significant peak at 650 Hz, where SAC values reach their maximum for the whole batch (~0.98). For configuration C1, the precision of the fit for the PICNN is excellent; it reproduces the experimental curve precisely throughout the whole frequency interval, including peak locations, their magnitude, and increased absorption above 1000 Hz. Thus, the more complicated the layers get, i.e., closer to a continuous layered porous medium, the better the acoustic transport equations in PICNN describe the propagation regime.

In configuration D, we see a totally different entangled structure that is chaotic and not symmetric geometrically, leading to a heterogeneous and anisotropic porous medium. It shows smooth broadband absorption without the presence of any clear peak and valley. In this case, the SAC shows a steady increment starting from 200 Hz, peaks around ~0.83 at 450 Hz, and remains very high (0.55–0.75) after that point. In this case, the PICNN model predictions are extremely accurate and almost identical to experimental values. They are even able to predict the small differences between experimental values in frequencies like 700 Hz and 1000 Hz. Unlike classical analytical models, for example, the Johnson-Champoux-Allard [58] model that requires detailed characterization of difficult to measure parameters, the PICNN incorporates the flexibility of a data-driven model along with energy and acoustic mass conservation.

A comparative analysis of the four graphs in Figure 9 allows us to draw important conclusions about the effectiveness of the PICNN approach in modeling the acoustics of unconventional materials such as bubble wrap:

1. The model offers its best performance in highly layered (Figure 9c) and randomly tangled (Figure 9d) configurations. In these cases, absorption is driven by diffuse dissipative phenomena that lend themselves well to being approximated by the neural network’s continuous activation functions.
2. The setup (Figure 9a) reveals the sole limitation of the model, which is the problem of simulating clearly defined and separated low-frequency resonance peaks together with valleys. This “artificial damping” effect is a well-known property during optimization of physics-informed neural networks and usually arises due to the ratio between data loss and differential equations loss (loss balancing).
3. In general, the capability of the PICNN to accurately simulate the behavior of SAC in cases of drastic change in the structure of the macrostructure (from ordered layer structures to completely tangled ones) without modification of the network structure shows that this instrument is a great alternative to the FEM and analytical approaches, saving time of calculation and enabling the geometric optimization of inexpensive sound absorbing materials.

#### 3.2.3. Quantitative Performance Evaluation

The predictive capability of the PICNN framework was assessed by means of four metrics that have been widely used for validating predictive approaches based on machine learning techniques: Root Mean Square Error (RMSE), Mean Absolute Error (MAE), Normalized Root Mean Square Error (NRMSE), and coefficient of determination (R^2^). RMSE is the measure of the standard deviation of the prediction errors and gives more sensitivity to the differences between the experimental and predicted values [59]. MAE is the average of the absolute value of the difference between the experimental and predicted SAC values, representing an indication of the prediction accuracy [60]. NRMSE was also included in order to provide a scale-free prediction accuracy measure. Contrary to the traditional measure of RMSE, which depends on the absolute magnitude of the investigated dataset, the NRMSE takes into account the normalized prediction error with respect to the variation range of the experimental SAC values, thus allowing a better comparison between different acoustic configurations [61]. Finally, the coefficient of determination R^2^ is used to quantify the correlation between predicted and experimental data sets [62].

Table 5 gives an overview of the Root Mean Square Error (RMSE), Mean Absolute Error (MAE), Normalized Root Mean Square Error (NRMSE), and the coefficient of determination (R^2^). The mentioned statistical measures are complementary to each other, giving a holistic picture of the prediction accuracy of the proposed model. The errors of the predictions and their agreement with the measured data are estimated.

The obtained results prove the high precision and stability of the constructed Physics-Informed Conditional Neural Network (PICNN) model for predicting sound absorption characteristics of bubble wrap geometries. In particular, the value of the coefficient of determination R^2^ = 0.9905 indicates high correlation between the experimental and theoretical data, with more than 99% of variation explained by the developed model. Low error parameters guarantee the reliability of the model: a Mean Absolute Error (MAE) of 0.0166 proves that there is a minor difference between the predicted and experimental SAC values; the Root Mean Square Error (RMSE) of 0.0242 confirms no discrepancies. It should be mentioned that the sound absorption coefficient is a dimensionless parameter from the range [0, 1]. Further evidence of the reliability of the assessment can be found in the normalized error measure, in which a value of 2.49% of NRMSE implies small prediction errors when compared to the entire range of SAC values obtained through experiments. The strong correlation together with low prediction errors is sufficient proof that the physics-informed neural network serves as a successful means of acoustically characterizing absorbers made from sustainable packaging. Through the utilization of physical knowledge about the relationship between geometry and acoustics, PICNN proves to have better generalization capabilities than purely empirical models.

#### 3.2.4. Correlation Analysis Between Experimental and Predicted SAC Values

**Figure 10:**
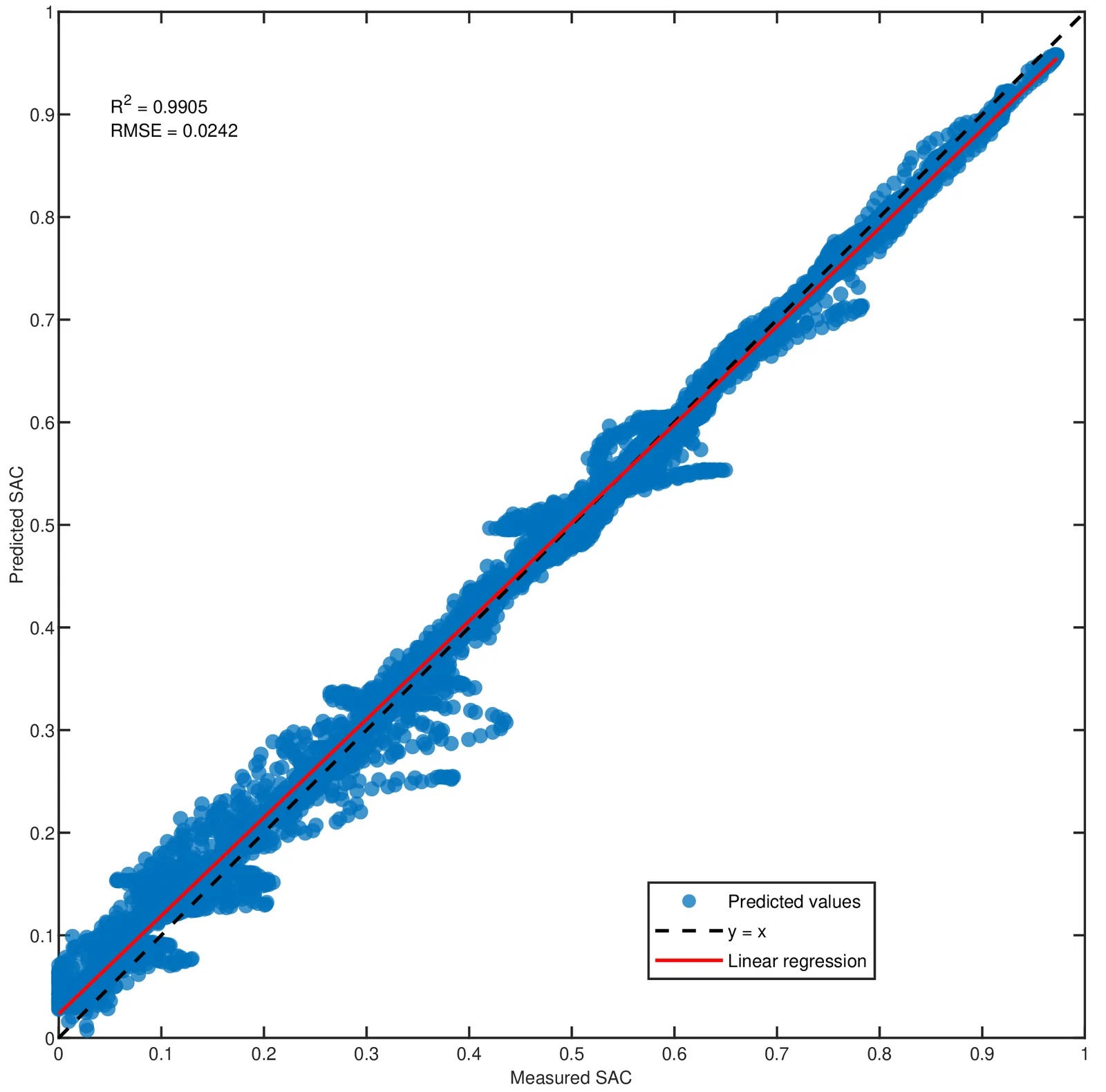
Measured SAC versus predicted SAC values based on the proposed Physics-Informed Conditional Neural Network (PICNN) model.

In order to analyze the predictive capability of the suggested PICNN-based model, the relationship between the experimentally determined SACs and SACs predicted by the proposed model was examined [63]. The graph representing the experimental data points for all bubble wrap configurations analyzed is presented in Figure 10. In this figure, the correlation line is y = x. The deviation of SACs predicted by the model from this line gives some idea of the prediction capability of the model. The correlation results shown in Figure 10 give additional confirmation of the predictive capability of the proposed Physics-Informed Conditional Neural Network (PICNN) model. Figure 9 illustrates the correlation between the predicted SAC values and their corresponding measured values for all bubble wrap configurations that have been analyzed. The fact that the data points lie on the ideal agreement line (y = x) indicates that there is a high level of correlation between numerical predictions and measured data. The small spread around the reference line indicates that there are no systematic errors due to overprediction or underprediction by the model. This becomes highly significant since we have to keep in mind the complexity of the considered configurations, consisting of different layering of bubble wrap, air cavities, and internal structures. The ability of the model to maintain the same level of prediction accuracy for the various configurations proves the robustness of the model and its ability to capture the physical relationships between the geometrical parameters and the acoustic properties.

The high correlation in the scatter plot is in agreement with the statistical measures presented earlier, specifically the coefficient of determination (R^2^ = 0.9905). The closeness of the regression line to the ideal line shows that the model captures almost all the variation in the experimental SAC data [64]. This shows that the physics-based constraints incorporated in the model successfully guided the learning process, reducing the risk of unrealistic predictions associated with data-driven models.

Moreover, the established match between experimentally found and predicted values stresses the possible effectiveness of the developed PICNN technique as a tool for predicting acoustic properties of materials. Contrary to traditional experimental techniques that imply the creation and evaluation of each particular setup, the suggested approach can be used to virtually optimize the acoustic absorbers made of bubble wrap by predicting their efficiency depending on design parameters. Thus, the performed correlation analysis proves the validity of the suggested approach and its applicability for developing environmentally friendly, inexpensive, and recyclable acoustic solutions for retrofits of residential buildings.

## 4. Conclusions

This experimental study demonstrates that reused bubble wrap, typically classified as low-grade packaging waste, can serve as an effective and tunable acoustic absorber when properly engineered. Analysis of seven distinct geometries showed that its acoustic performance can be tuned across a broad frequency range by varying the number of layers, cavity depth and shape, and structural configuration. Bubble wrap should not be treated as a simple polymeric membrane but as a flexible acoustic medium whose performance strongly depends on geometry. As a stretched membrane over an air cavity, it exploits resonant effects between the lightweight film and the trapped air volume: deepening the backing cavity shifts resonance frequencies lower, improving absorption in the low-frequency range relevant to building acoustics. As the samples were taken directly from the packaging waste and have not been pre-treated prior to testing, the possibility of contaminant residue may be viewed as an uncontrolled variable that influences the resulting acoustic performance. Additional research involving treatment of the samples and other waste flows will be beneficial for evaluation of this variable’s influence.

Multilayering further enhances performance by increasing surface mass and dissipative interfaces. Double- and triple-layer samples outperformed single-layer structures, with the double-layer configuration (6 cm total cavity depth) reaching a peak absorption coefficient of 0.91 near 640 Hz, and the triple-layer sample achieving a wider absorption bandwidth across the mid-frequency range. This confirms that multilayer designs can improve performance without additional materials, using only recovered packaging. A honeycomb arrangement behind each layer yielded an additional advantage in that although the absorption was not maximized to the same extent as in the analogous air-cavity arrangement, the resonance effect became smoothed out and the absorption more uniform over the frequency range, as a result of higher losses due to viscosity and heat transfer associated with the flow resistance of the honeycomb.

Most important is the behavior of the bubble wrap in the rolled configuration, which represents a heterogeneous porous material and not a resonant absorber. The complex pore structure allows for constant energy dissipation through the thickness of the material, resulting in broadband absorption coefficients above 0.50 and reaching up to 0.83 near 440 Hz without any dips, which are typically present in membrane-like materials. In addition to its superior absorption properties, the rolled configuration features the simplicity of manufacturing that does not involve any frame, spacers, or geometrical optimization. These observations determine two distinct design concepts: membrane configurations for frequency-selective absorption and resonator control, and the rolled configuration for broadband absorption.

In addition to the experimental analysis, the suggested Physics-Informed Conditional Neural Network (PICNN) successfully replicates the experimental results with the same high precision (R^2^ = 0.9905, MAE = 0.0166, RMSE = 0.0242, NRMSE = 2.49%). In other words, incorporating physical constraints increased the prediction accuracy in comparison with the data-driven approaches, which means that the PICNN can be applied for fast and cost-efficient optimization of the new bubble wrap geometries without performing numerous physical tests.

From the point of view of reuse of materials and resourcefulness, the significance of the obtained results could be assessed. Bubble wrap is used extensively in plastic packaging and could be regarded as a representative example of post-consumer waste. The results of the current research show that bubble wrap could serve as an effective acoustic absorber depending on the configuration. Thus, it is possible to assume that the material could be used as an acoustics absorber without any special production procedure. Its light weight, flexibility, waterproofing, and availability might make bubble wrap an interesting material for temporary or light acoustic solutions and other purposes for which these properties are needed. Nevertheless, the present research could hardly be considered a full-scale evaluation of the sustainability of the material. Such characteristics as durability, aging, fire safety, hygiene, economic efficiency, and environmental aspects of life cycle have not been taken into account.

It is necessary to list some limitations. In this study, only normal-incidence absorption in the impedance tube was evaluated, and further study must consider random-incidence absorption in reverberation chambers, as well as airborne sound insulation, mechanical robustness, fire and water resistance, aging effects, and the environmental impact throughout its life cycle. First of all, it must be stated that this experiment was not a full factorial one, and therefore the quantitative effect of each of the studied parameters cannot be estimated. Also, there were no control experiments for individual elements of the structure: empty cavity, plain LDPE film, and core alone. As a result, the obtained acoustic characteristics can be referred to the studied configurations only, while the contribution of individual components cannot be estimated separately. Moreover, the described acoustic phenomena of viscous, thermoacoustic, and viscoelastic nature are not confirmed by any independent measurements, such as airflow resistivity and surface impedance. Modeling in the future might involve combining transfer matrix techniques, finite element modeling, and topology optimization with the concept of the PICNN model to design optimized multi-layer acoustic metamaterials made of repurposed packaging materials. A comparison study involving ablative analysis between the PICNN and the corresponding conventional neural network will also shed light on the role and generalization ability of the physics-informed modelling approach. In summary, it is clear that used bubble wrap can be considered an acoustic material that has high efficiency, is lightweight, affordable, and even has an environmental advantage due to physics-informed modelling. Therefore, this material should be considered a reusable material rather than a disposable one in building acoustic systems.

## Figures and Tables

**Table 1:** Properties of the low-density polyethylene (LDPE) used in bubble wrap.

Property	Value	Unit	Property	Value	Unit
Chemical formula	(C_2_H_4_)_n_	—	Maximum operating temperature	80–105	°C
Density	0.91–0.94	g/cm^3^	Glass transition temperature (Tg)	−125	°C
Young’s modulus	200–300	MPa	Crystallinity	50–60	%
Tensile strength	8–21	MPa	Thermal conductivity	0.33–0.35	W/(m·K)
Elongation at break	100–650	%	Chemical resistance to diluted acids/bases	Elevata	—
Shore D hardness	41–46	—	Water vapor permeability	Bassa	—
Melting temperature	105–115	°C	Recycling code	4 (LDPE)	—

**Table 2:** Performance comparison across the evaluated sound-absorbing configurations.

Configuration	Structure Type	Total Thickness	Main Peak (f_0_, SAC)	Dominant Spectral Behavior
A2 (1 Layer)	1 layer + 6 cm cavity	6 cm	680 Hz SAC ≈ 0.78	Narrowband resonance, rapid decay
B2 (2 Layer)	2 layers + subdivided cavities	6 cm	640 Hz SAC ≈ 0.91	High efficiency at medium frequencies, coupled resonance
C1 (3 Layer)	3 layers + 2 cm cavity each	6 cm	660 Hz SAC ≈ 0.97	Maximum peak absorption (near-unity)
D (Full)	Ball/rolled bulk	6 cm	440 Hz SAC ≈ 0.83	Extended broadband, shift to low frequencies

**Table 3:** Table of summary sound absorption indicators.

Configuration	NRC	SAA	Average SAC (200–1500 Hz)	Average SAC (200–500 Hz)	Average SAC (500–1000 Hz)	Average SAC (1000–1500 Hz)
A1	0.10	0.11	0.236	0.057	0.127	0.452
A2	0.25	0.29	0.37	0.15	0.493	0.378
B1	0.30	0.31	0.472	0.126	0.512	0.64
B2	0.30	0.39	0.486	0.212	0.633	0.503
C1	0.30	0.39	0.496	0.239	0.678	0.467
C2	0.45	0.48	0.623	0.268	0.801	0.658
D	0.50	0.55	0.611	0.545	0.665	0.597

**Table 4:** Training parameters and neural network configuration.

Parameter	Value	Parameter	Value
Initial learning rate	0.001	Output layer	1 neuron, linear
Maximum epochs	500	Input normalization	Z-score
Batch strategy	Full batch	Physics-informed weight (*λphys*)	0.1
Activation function	Tanh	Boundary-loss weight (λbound)	Not used
Hidden layers	3 (64–64–32 neurons)	Early stopping	No

**Table 5:** Performance statistical indicators of the PICNN model for the prediction of the sound absorption coefficient (SAC) of the bubble wrap configurations under consideration (RMSE, MAE, NRMSE, R^2^).

MAE	RMSE	NRMSE	R^2^
0.0166	0.0242	2.49%	0.9905

## Data Availability

The original contributions presented in the study are included in the article/Supplementary Materials, further inquiries can be directed to the corresponding author.

## Supplementary Materials

The following supporting information can be downloaded at: https://www.mdpi.com/article/10.3390/polym18202460/s1, Supplementary Material: Standard Deviation (SD) per Frequency.
